# Radiofrequency Energy Harvesting Systems for Internet of Things Applications: A Comprehensive Overview of Design Issues

**DOI:** 10.3390/s22218088

**Published:** 2022-10-22

**Authors:** Alex Mouapi

**Affiliations:** Department of Industrial Electronics at the Cegep of Abitibi-Temiscamingue, 425 Blvd. du Collège, Rouyn-Noranda, QC J9X5E5, Canada; alex.mouapi@cegepat.qc.ca

**Keywords:** RF-EH-WS, MEB-WS, rectenna, PMM, energy efficiency, RF power level, RFT, IoT

## Abstract

Radiofrequency energy harvesting (RF-EH) solutions have evolved significantly in recent years due to the ubiquity of electromagnetic waves in any environment. This review presents a comprehensive report on autonomous wireless sensor (WS) design considerations based on RF-EH. The obtainability of RF-EH-WS is driven by development efforts in the areas of RF-EH circuit design, known as rectifying antenna (Rectenna), the minimization of the energy budget of WS (MEB-WS), and finally, power management modules (PMM). The PMM aims to optimize the energy efficiency of the WS. In addition to these three factors, examining the RF power levels harvested related to the rectenna feeding technique (RFT) is essential. Since we did not find any review presenting a holistic view of these design considerations, we strived to provide a detailed picture of recent advances and new enhancements in this review. To address this issue, this review gives an overview of the seminal and contemporary studies in the RF-EH-WS field. The IoT issues are also discussed in terms of their basic requirement to support reduced size or miniaturized smart objects, which are common matters in current applications of WS nodes. Potential open issues that might be considered for future research are also discussed in this article. For a more detailed description of all presented concepts, many significant references are provided for the readers.

## 1. Introduction

Internet of Things (IoT) implies a set of infrastructures and technologies. This is to ensure, through a network, the connection of physical objects to enable them to exchange information about themselves and their environment. IoT refers to a network in which objects, animals, and people have unique identifiers and can transfer data without requiring human-to-human or human-to-machine interaction [1]. Identifying all IoT applications is not the purpose of this review, but IoT applications are numerous, and some leading objectives are smart buildings, smart cities, industry 4.0, etc. [2]. Smart cities, for example, involve intelligent monitoring and management of transport systems, smart hospitals, structural health monitoring systems, etc. Smart buildings include many sensors to monitor environmental data such as temperature, brightness, humidity, etc. This does not only preserve the environment but also ensures the comfort of the users. In the industrial sector, IoT enables intelligent automation and the integration of new technologies in various processes to improve productivity, safety, and quality of service [3].

This global view of the main applications of the IoT shows that the objective is to support real-time communication to monitor and act on physical processes. Additionally, systems communicate with each other and humans to decentralize decision-making. The IoT paradigm emerges from developments in several research fields: sensor networks, the web, and cloud computing [4]. Unlike other elements that can be placed in easily accessible locations, the sensor can be found in the walls of buildings, the deep ocean, inhospitable terrain, and even battlefields. The sensors can be wired or wireless. Wireless sensors (WS) are more attractive because they offer more flexibility in their deployment [5].

Since they are battery-powered, recharging the WS’s battery is expensive or even impossible under certain conditions. To extend the lifespan of WSs, a new research field has evolved in recent years, known as energy harvesting (EH). These WSs are now well known in the literature as EH-WS [6]. An EH process involves identifying a primary energy source in the WS vicinity and converting it into electrical energy directly usable by the WS. This possibility of EH-WSs is made feasible by combining two research fields: the first field maximizes the performance of energy harvesting circuits, while the second minimizes the energy budget spent on WSs (MEB-WS). Notably, both research fields have tended to evolve separately and independently.

In ambient EH, the proposed techniques rely entirely on the primary energy sources. The main sources are airflow, internal light, thermoelectric gradient, vibration, and radiofrequency (RF) energy. For a long time, comparing primary energy sources was based on the power density of the harvesting system [7]. Today, the need to deploy WS in environments that are difficult to access requires having a primary source that can be available everywhere. Among the primary sources cited above, radiofrequency waves offer this flexibility. For this reason, this review proposes the design considerations of autonomous WSs powered by an RF energy harvesting system to support the objectives of IoT devices and networks. The WS thus defined will be referred to throughout this paper by RF-EH-WS. In the following subsections, we briefly describe these considerations and our study problem.

### 1.1. Design Considerations of the RF-EH-WS

As mentioned earlier, the RF-EH-WS derives its existence from the ubiquity of RF sources and joint efforts in the fields of RF-EH and MEB-WS. To stimulate new optimal solutions for RF-EH-WS, we review the optimizations proposed individually in recent years in these three research fields. When it comes to RF-EH systems, the main method used is an *antenna* to capture the ubiquitous radio frequency waves. An *RF/DC converter* is then used to format the harvested energy. A *maximum power point tracking* (MPPT) circuit based on a DC/DC converter is required to transfer the maximum power to the WS. A *matching filter* must be inserted between the antenna and RF/DC converter to reduce energy loss by reflection. The system thus constituted is called a rectenna for rectifying antenna.

The overall performance of the rectenna depends on the amount of RF energy picked up by the antenna. This energy can be generated artificially; in this case, it is a wireless power transfer (*WPT*). The rectenna can also be designed to be powered by ambient RF energy available due to the operation of other telecommunications equipment not included in the harvest chain. The latter case is known as ambient RF energy harvesting (*A-RF-EH*). Considering that an inherent feature of RF energy involves the utilization of a WS antenna both to transmit data and collect energy, the current trend in RF-EH-WS is to support both wireless information and power transmission (WIPT) concepts. Three main areas of WIPT have been identified [8]: wireless powered communication network (*WPCN*), wirelessly powered backscatter communication (*WPBC*), and simultaneous wireless information and power transfer (*SWIPT*). These rectenna feeding techniques will be described in Section 3 of this paper.

An overview of all the solutions proposed in recent years in the field of MEB-WS makes it possible to target three main factors that influence the consumption of WS. These are the *hardware* that constitutes it, *network topology*, and *communication protocol*.

**Figure 1:**
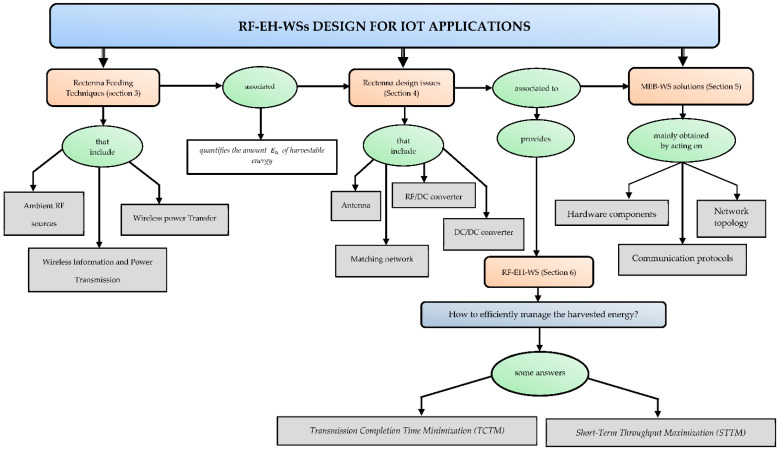
RF-EH-WS design considerations and paper organization.

By pooling these three fields of research (RF feeding techniques, RF-EH, and MEB-WS), the problem arose of the efficient management of the energy harvested. Thus, the operability of an RF-EH-WS results from four main topics: rectenna feeding techniques (RFT), RF-EH (Rectenna), MEB-WS, and the efficient management of the harvested energy. These four fields of research are represented in Figure 1, which shows the conceptual map of this study. The block that deals with RF-EH-WS is known as the power management module (PMM) [9]. In a real IoT network, events can occur at any time. If the harvested energy level is not enough to transmit them, these will differ later. This data storage then induces a delay in data transmission. The primary function of the PMM module is to minimize differed transmission delays. To achieve this goal, two main problems are defined in the literature, namely: *transmission completion time minimization* *(TCTM)* [10] and *short-term throughput maximization (STTM)* [11]. These two leading solutions for optimizing the energy efficiency of RF-EH-WS will be detailed in Section 6 of this paper.

### 1.2. Problematic and Contributions

In most literature overviews proposed in recent years, authors are only interested in one or two parts of the diagram shown in Figure 1. In [9], for example, Ibrahim et al. proposed a comprehensive review of the design and methodologies of the RF-EH system. While offering a set of potential IoT applications, the authors are not interested in MEB-WS solutions that are known to help optimize the performance of an RF-EH-WS. Cansiz et al. proposed in [12] a comprehensive review dealing only with efficiency in RF-EH systems. More specifically, the receiving antenna’s characteristics and diode characteristics’ impact on the RF/DC conversion efficiency were discussed in this previous study. Regarding RFT, only the case of ambient RF energy harvesting has been considered. Wireless power transfer solutions have not been mentioned. Sleebi et al. propose in [13] the review entitled “RF Energy Harvesting: An Overview and Design Issues”. An emphasis is placed only on the design considerations of the receiving antenna and those of the RF/DC converter. The contributions of the MPPT circuit and solutions for managing the harvested energy have not been proposed.

This summary view of some reviews proposed in recent years shows that very few studies offer a comprehensive view of the considerations for achieving the operability of an RF-EH-WS in a real environment. This is the problem of our review, which is organized around the four research axes mentioned above: RFT, RF-EH, MEB-WS, and PMM. It is, therefore, these four main topics that will guide the writing of this paper with the following objectives:

- Define the features of the main components of an RF-EH-WS.
- Provide design considerations and efficiency analysis of RF/DC conversion systems.
- Compare the performance of different circuit topologies.
- Review the leading solutions for the MEB-WS.
- Remind readers of the theoretical foundation through design equations.
- Define future research for the RF-EH-WS.

This review is thus intended for both RF harvester circuit designers and communication protocol developers to enable new solutions adapted to RF energy harvesting systems for WS. This paper reviews the fundamental principles through design equations and optimization solutions for each of the points mentioned above. In addition to these main topics, a comparison with existing related reviews will be proposed in Section 2. The rest of the paper will be organized as follows: Section 3 deals with rectenna feeding techniques. Section 4 presents recent progress in RF energy harvesting systems; it addresses modeling and optimization issues of rectennas. In Section 5, solutions for the MEB-WS are discussed. Section 6 reports proposed solutions for efficiently managing the harvested energy in an RF-EH-WS. In Section 7, new RF-EH-WS design schemes based on compatible solutions for IoT applications are offered as areas of future research. Finally, Section 8 concludes this review.

## 2. Comparison with Related Reviews

Few existing reviews offer a global view of the literature covering the topics mentioned in the introduction. Some directly propose solutions to manage the energy harvested without being interested in the processes of transforming RF waves into DC electrical energy suitable for powering the WS [14,15]. Other studies deal only with RF/DC transformation without examining rectenna powering techniques [16,17]. The solutions for minimizing WSs’ energy budget are often ignored or treated independently. Other studies related to ours are proposed in [15,18,19,20,21,22,23,24,25,26]; a summary of the key points developed in these related studies is proposed in Table 1.

In [14], Kansal et al. proposed a set of algorithms for efficient management in the EH-WS. The construction of algorithms is focused primarily on observing the residual energy of the battery. This previous study does not address the design of the harvesting circuits. In [15], Sujesha et al. proposed an investigation into the implications of the EH-WS. The case of RF and solar sources was presented from the point of view of energy management. Specifically, the issue of predicting harvestable energy using an exponentially weighted moving-average (EWMA) filter was discussed. However, as in [14], energy conversion circuits were not examined. In [21], Valenta and Durgin comprehensively study WPT systems in which specific optimization of the rectenna’s circuits is developed. Emphasis is placed on the characteristics of the rectifier diode and impedance matching methods. However, the solutions for managing the harvested energy are not investigated. In [18], Lu et al. discuss wireless networks with RF energy harvesting capability. This review presents a global overview of the RF energy harvesting chain, focusing specifically on different types of wireless networks, namely single-hop networks, multi-antenna networks, relay networks, and cognitive radio networks. However, this previous study does not examine solutions for minimizing energy dissipation in WS. Ku et al. have proposed in [19] the past, present, and future challenges of energy harvesting communications. Emphasis is placed on the randomness of the amount of harvestable energy. Deterministic and stochastic models of EH have been presented. Unfortunately, the architecture and harvesting circuits’ different topologies are not dealt with. In [22], particular interest is devoted by Mohjazi et al. to designing cognitive radio networks powered by RF energy. Only two techniques (WPT and A-RF-EH) for feeding the rectenna are mentioned. Emphasis is also placed on energy management through throughput maximization. However, no information is provided on the hardware architecture of RF transmitters and receivers. In [17], Soyata et al. offer an overview of RF energy harvesting for embedded systems. The authors proposed a study on the architecture of the RF-EH-WS and ways of improving signal transmission. In [20], Tharindu et al. summarize the recent progress made in SWIPT, and from that, they define future challenges. After a brief history and classifying the various WPT techniques, they present the receivers’ architecture in the SWIPT. In [27], Sidhu et al. investigated the various ambient RF sources that can be exploited to power the rectennas. Surrender et al. have provided a review of rectenna design strategies for wireless applications in [26]. Applications covered include wireless sensor networks, wireless power transfer systems, solar energy transmission, medical implants, and wireless energy harvesting. A detailed description of the blocks of a rectenna is provided in this recent review. Then, the authors focused on the main techniques allowing antennas with circular polarization, which makes it possible to maintain constant output performances. In [9], Ibrahim et al. offered a comprehensive review on the design of radiofrequency energy harvesting technologies. The different topologies of energy transformation circuits were detailed. However, energy management protocols were not discussed. Performance metrics of RF-EH systems, as well as power management protocols, have been discussed by Hafiz et al. in [25]. However, MEB-WS solutions have not been addressed.

## 3. Rectenna Feeding Techniques (RFT)

Regardless of the optimizations made in the circuit design of an RF-EH-WS, the overall performance of such a system will still depend on the amount of energy that can be harvested. One of the first design steps is assessing the harvestable energy potential in the environment where the sensor will be deployed. We have referred to this design consideration as rectenna feeding techniques (RFT) in this review. It will then be a question of presenting the leading solutions commonly considered.

One of the first RFT solutions considered was to recycle part of the radio frequency waves available in the environment due to the operation of telecommunications devices. This solution is known as ambient radiofrequency energy harvesting (A-RF-EH). Recently, campaigns to measure power density at different frequencies have been proposed [27,28]. One of the limitations of this feeding technique was then the low levels of power densities freely emitted. A second RFT method uses a dedicated source to power the WS. In this case, an emitting source (antenna) of RF waves is used to power the rectenna; the harvestable power is evaluated through various RF propagation models that differ depending on the transmission channel between the emitting source and rectenna. This RFT is known as wireless power transfer (WPT). Since the WPT is done via an antenna, and the WS also uses an antenna to transmit data taken from the environment, the current WS trend is to support wireless information and power transmission (WIPT) concepts [8]. Three leading solutions are proposed; these are wireless powered communication networks (WPCN) [29], wirelessly powered backscatter communication (WPBC) [30], and simultaneous wireless information and power transfer (SWIPT) [20]. The main RFTs presented above will be described in estimating the amount of harvested power in the following subsections.

### 3.1. Ambient RF Energy Harvesting (A-RF-EH)

The A-RF-EH aims to recycle energy available in the environment from the surrounding activity of wireless communication devices. The frequency bands commonly considered are those of digital TV (DTV), 3G, LTE (long term evolution), the global system for mobile (GSM) band, and Wi-Fi (wireless fidelity). Table 2 presents some power densities found in the literature.

The data in Table 2 above clearly show that the naturally available RF power levels in the environment are too low. However, several designers have been able to propose solutions for harvesting usable quantities of power. These solutions rely mainly on the design of circuits capable of simultaneously gathering RF energy through several frequency bands [28]. It should be recalled that those circuits provide the best performances at the expense of congestion. This is unsuitable for most applications because congestion is one of the design constraints.

### 3.2. Wireless Power Transfer (WPT)

WPT can be done in two ways: by exploiting the coils’ magnetic field to transport electrical energy or using antennas coupled to an RF wave emitting source. The WPT with coils was initially proposed by Nicolas Tesla and exploited the principle of magnetic resonance of two coils to transport large amounts of energy to places distant from the power source [36]. This WPT technique is used in many applications such as biomedical devices [37] and radio frequency identification (RFID) chips [38]; its main drawback is its limited transmission range. In addition to this low transmission range, the power levels are very high, leading to potential health concerns. For example, in [39], it was possible to transfer 60 W with a conversion efficiency of 40% at only 2 m. This technique of WPT is known as near-field transmission [37].

**Figure 2:**
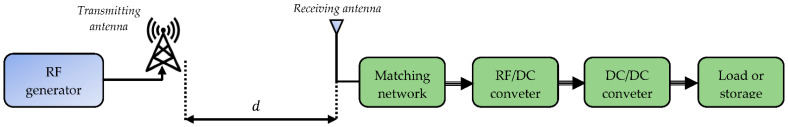
Far-field WPT and RF/DC conversion blocks of an RF-EH system.

The far-field technique in which power is transferred by electromagnetic radiation is increasingly considered to transfer energy wirelessly at greater distances. As shown in Figure 2, the system thus consists of an RF generator emitting a power $P_{T}$. The generator is coupled to a transmitting antenna with gain $G_{T},$ which radiates electromagnetic waves. One or more receiving antennas with gain $G_{R}$ located at a distance d from the transmitting antenna make it possible to capture the radiated RF energy. An RF/DC conversion is then necessary to shape the energy. Between the converter and the reception antenna(s), a matching network makes it possible to reduce losses by reflection between the antenna and RF/DC converter. Finally, a DC/DC converter must be used to match the energy to the load or the storage element.

Knowing the emitted power $P_{T}$, it is essential to evaluate the received power $P_{R}$ to make a judicious choice of the other components of the system. The harvestable power depends mainly on the distance d between the antennas, their gain, and the frequency of the transmitted signal. The basic model commonly considered is the Friis model, which is used when the transmitting and receiving antennae are in an empty environment without obstacles (line-of-sight environments). In this case, the power received is expressed as follows:

(1) $$P_{R}=P_{T}G_{T}G_{R}{\left(\frac{c}{4\pi df}\right)}^{2}$$

where c is the speed of light and f is the emission frequency of RF waves. The other parameters are defined as above.

The Friis model was used in [40] to evaluate the power harvested by a rectenna fed by several emitting sources. Assuming N identical energy sources emitting the same power, $P_{T}$ with the same transmission gain $G_{T}$ at the same frequency f, Naderi et al. showed that the received power is defined as follow:

(2) $$P_{R}=P_{T}G_{T}G_{R}{\left(\frac{c}{4\pi f}\right)}^{2}\left[\overset{N}{\underset{i=1}{\sum }}\frac{1}{d_{i}^{2}}+\overset{N}{\underset{\begin{array}{c} i=1\\ i\neq j \end{array}}{\sum }}\overset{N}{\underset{j=1}{\sum }}\frac{\cos\left(k\left(∆d_{ij}\right)\right)}{d_{i}d_{j}}\right]$$

where k is the wavenumber, $d_{i}$ is the distance between the receiving antenna and transmitter i, $∆d_{ij}=\left|d_{i}-d_{j}\right|$ is the distance separating the receiving antenna from both emitters i and j. The term $k\left(∆d_{ij}\right)$ represents the phase difference between the two signals. It makes it possible to model constructive or destructive interference. Using Equation (2), the authors defined optimal positions of the emitting sources to avoid destructive interferences.

To improve the accuracy in estimating the harvestable power, considerations of reflection, diffraction, and scattering of the waves must be considered. In this case, the power is evaluated as in Equation (3), and the model is known as the two-ray model [41].

(3) $$P_{R}=P_{T}G_{R}G_{T}\frac{h_{T}^{2}h_{R}^{2}}{d^{4}}$$

where $h_{R}$ and $h_{T}$ represent the effective height of the receiving and transmitting antenna, respectively; the other parameters are defined as above.

To estimate the harvestable power in various non-line-of-sight environments, many models derived from a combination of analytical and empirical methods have also been established. One of the most popular is the log-distance path-loss model; this includes random shadowing effects caused by a signal obstruction such as a building. The harvestable power is, in this case, expressed as follows [41]:

(4) $$P_{R}\left(d,n\right)=P_{R}\left(d_{0}\right){\left(\frac{d_{0}}{d}\right)}^{n}$$

$P_{R}\left(d_{0}\right)$ is the received power at the $d_{0}$ distance, which is a reference distance, and n is the path loss exponent. The value of n always relates to the propagation environment features. For instance, in [42], the value of 1.6 is reported for an office building.

In contrast to previous deterministic models, probabilistic models offering more realistic modeling have also been proposed. The most used probabilistic model is that of Rayleigh, which represents the scenarios in which there is no line of sight between the transmitting and receiving antennas. In the Rayleigh model, the harvestable power is expressed as follows [43]:

(5) $$P_{R}=P_{R}^{\det}\times 10^{\left(n.\log_{10}\left(d/d_{0}\right)\right)}\times {\left|r\right|}^{2}$$

where $P_{R}^{\det}$ represents the received power estimated by deterministic models, n is the path loss factor exponent, d is the distance between the receiving and transmitting antenna, $d_{0}$ is a reference distance, and r denotes a random number following complex Gaussian distribution.

The above presents four RF propagation models commonly used in far-field WPTs. In what follows, we present how the harvestable power is evaluated in the case of wireless information and power transfer.

### 3.3. Harvestable Power in Wireless Information and Power Transfer (WIPT) Techniques

**Figure 3:**
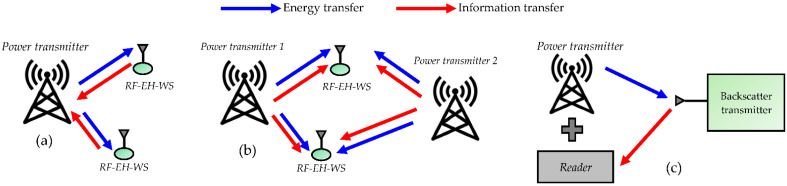
Various WIPT architectures. (**a**) WPCN. (**b**) SWIPT. (**c**) WPBC.

A WIPT system exploits RF waves to transfer energy and information in a wireless sensor network. In such systems, the design issues include information power separation techniques and how the signals should be designed to achieve the best compromise between the amount of information and amount of energy that can be transferred. Three leading solutions whose different architectures are shown in Figure 3 are considered in the literature [8].

- In the wireless powered communications network (WPCN), shown in Figure 3a, the WSs harvest RF energy, then use that energy to actively transmit data.
- In simultaneous wireless information and power transfer (SWIPT), shown in Figure 3b, energy and information are simultaneously transferred from one or more BS to one or more WSs. The WS can then choose to decode information or harvest energy sent from the power transmitter by switching between the decoding and harvesting modules to achieve high efficiency of energy-information transmission.
- In wirelessly powered backscatter communications (WPBC), shown in Figure 3c, a backscatter device modulates and reflects an RF signal instead of generating a new signal; the backscattered power is intended to supply the reader.

As shown in the different architectures in Figure 3, the models considered for estimating the harvestable power used in WPTs are valid for evaluating the autonomy of WPCNs. In the following, we propose quantifying the harvestable power in the case of SWIPT and WPBC.

#### 3.3.1. Harvestable Power in Simultaneous Wireless Information and Power Transfer (SWIPT)

**Figure 4:**
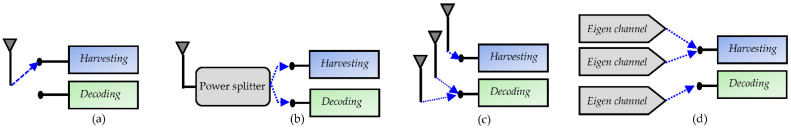
SWIPT transmission techniques in different domains: (**a**) Time. (**b**) Power. (**c**) Antenna. (**d**) Space.

The SWIPT concept was initially introduced in [44], while the recent enhancements were proposed in [20]. Those studies recall some basic principles and suggest a few alternatives to optimize the efficiency of the RF-EH-WS. Initially, the precursors of the SWIPT advocated that the same signal could transmit energy and information without any loss [44]. Although there are fundamental compromises between information and power transfer, it is still not easy to implement in practice since the energy harvesting process destroys the information content. The harvestable power will then depend on the technique for separating coded information from the RF energy harvesting process. The four main techniques (shown in Figure 4) are: time switching, power splitting, antenna switching, and spatial switching techniques [20,45].

1. Time switching implementation

Figure 4a shows that the same antenna is used for the RF-EH and information decoding (ID). A switch changes the sensor’s reception type from RF-EH to ID or vice versa. The separation of the signal is done in the time domain. Denoting by T the total transmission time, a fraction τT is used for the power transfer, and the other fraction (1−τ)T for the information delivery. τ∈]0,1[ is an important design parameter called the time switching coefficient. When the sensor operates in RF-EH mode, the amount $E_{TS}$ of the energy harvested is defined by:

(6) $$E_{TS}=\tau T\eta P_{T}{\left|h\right|}^{2}$$

where η is the conversion efficiency of the rectenna; it will be discussed in the following section. h is the complex channel gain between the transmitter and receiver, and finally $P_{T}$ is the transmitted power.

2. Power splitting

As shown in Figure 4b, the input signal is divided into two power streams instantly dedicated to ID and RF-EH modes, making it possible to achieve both modes simultaneously. The ratio devoted to each process can be individually optimized. If θ ∈]0,1[ is the signal fraction used for RF-EH, as in the time switching architecture, the energy $E_{PS}$ harvested by the receiver can be calculated as follows [46]:

(7) $$E_{PS}=\theta T\eta P_{T}{\left|h\right|}^{2}$$

θ is the critical parameter of this design, also called the power splitting factor. 

The power splitting architecture, allowing instantaneous SWIPT, is better suited for applications with time constraints. Moreover, it has been theoretically established in [47] that the power splitting technique achieves better tradeoffs between information rate and amount of RF-EH. However, recently in [48], it has been shown that at low levels of the signal-noise ratio, the throughput of an architecture based on the time splitting technique is higher than that obtained with a PS architecture.

3. Antenna Switching

The antenna switching architecture, shown in Figure 4c, uses several antennas that are always divided into two groups. When one group is assigned to the information mode, the other is allocated to the RF-EH. The SWIPT is done here on the antenna field. This technique proposes optimization problems to determine an optimal number of allocated antenna elements to support a given communication frame [49]. Moreover, with an appropriate antenna switching protocol, it is possible to increase the system’s performance by increasing the number of antennas [20]. However, the antenna switching technique’s optimal performance increases the system’s complexity because of its architecture.

4. Spatial Switching

As shown in Figure 4d, the SWIPT is carried out in the space domain by exploiting several degrees of freedom of the interference channel. The sensor also has several antennas [50]. The communication channel is considered a multiple input multiple output (MIMO) channel subdivided into many channels that can convey either information or RF-EH. Any optimization in the spatial switching technique is challenging to achieve. This is mainly because channel assignment and power allocation issues involve complex nonlinear combinatorial optimization.

#### 3.3.2. Harvestable Power in Wirelessly Powered Backscatter Communications (WPBC)

**Figure 5:**
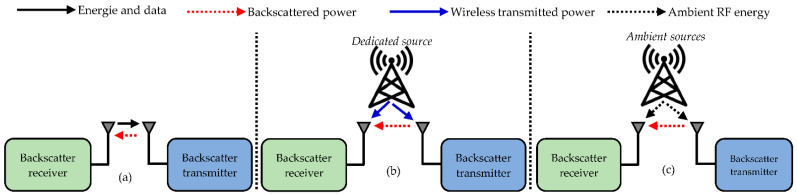
Different types of backscatter communications systems. (**a**) Monostatic. (**b**) Bistatic. (**c**) Ambient.

The energy is harvested, in this case, from the backscatter of an original signal; the backscatter being the reflection by a diffuser of the electromagnetic waves in directions opposite to the direction of arrival. As in the case of SWIPT, different architectures of WPBC exist. Three main techniques can be distinguished, which are shown in Figure 5. The architectures differ from each other by the source of the original signal. As shown in this figure, monostatic backscatter is distinguished from bistatic and ambient backscatter [51].

- In the monostatic systems shown in Figure 5a, the original signal comes from the backscatter receiver. The transmitted signal contains both energy and information. The backscatter transmitter radiates some of the energy to power the receiver. It is this technique that is used in RFID systems.
- In the bistatic systems shown in Figure 5b, the original signal transmitter differs from the backscatter receiver. The latter receives its energy from a dedicated source and the backscattered signal.
- In ambient backscatter in Figure 5c, the original signal comes from the ambient energy available due to the operation of telecommunications devices (digital TV, Wi-Fi, etc.). This energy is used to power both the backscattered transmitter and backscattered receiver. The backscattered energy thus makes it possible to increase the energy autonomy of the backscatter receiver

Regardless of WPBC’s category, it is evident that the strength of the backscattered signal is much lower than that of the original signal since it travels a much higher distance. If an original power $P_{T}$ is emitted, then the received power $P_{R}$ can be evaluated by considering the path loss between the power transmitter and backscatter transmitter, losses in the backscatter transmitter, and finally, path loss between the backscatter transmitter and backscatter receiver. Taking into account the three losses above and using the Friis transmission, the power received $P_{R}$ is estimated as follows [52]:

(8) $$P_{R}=P_{T}.G_{T}.G_{R}.G_{BT_{t}}.G_{BT_{r}}{\left(\frac{c^{2}}{16\pi^{2}f^{2}d_{1}d_{2}}\right)}^{2}$$

where $P_{T}$ represents the transmitted power; $G_{T}$ is the gain of the power transmitter; $G_{R}$ is the gain of the backscatter receiver; $G_{BT_{t}}$ and $G_{BT_{r}}$ are, respectively, the gains of the backscatter transmitter from the perspective of the transmit and receive antennas; $d_{1}$ and $d_{2}$ are, respectively, the distances between the power transmitter and backscatter transmitter and that between the backscatter transmitter and the backscatter receiver; c is the speed of light; and finally, f is the frequency of the transmitted signal.

All the above summarizes the main feeding techniques of rectennas. For each of the methods, we reviewed the models proposed in the literature. These models must be considered before circuit design because they make it possible to estimate the amount of the harvestable energy on which the circuit’s performance depends. The following section presents the design steps of RF/DC converters (rectenna).

5. Rectenna Design Issues

The different design steps of RF/DC converters are shown in Figure 2. The first element of the chain is the transducer (the antenna), which oversees capturing an RF power whose level is estimable by one of the feeding methods mentioned below. Then, an RF/DC converter, usually based on Schottky diodes, is used for signal shaping. The third design step is the matching network that minimizes reflection losses. Finally, a DC/DC converter is needed to adapt the energy to the load or storage element. The following subsections discuss the design considerations of these different blocks. More specifically, it will be a question of defining the characteristics of each of the blocks while highlighting the elements that impact their interdependence.

## 4. Rectenna Design Issues

### 4.1. The Receiving Antenna

The receiving antenna is one of the essential elements of the chain since the performance of the rectenna is entirely a slave to the characteristics of the receiving antenna. The general characteristics of the antenna will first be presented, followed by a review of the solutions proposed to achieve usable power levels.

#### 4.1.1. Main Features of the Receiving Antenna

The overall performance of the RF-EH system will depend on the radiation characteristics of the receiving antenna, the main ones being: the operating frequency, gain, radiation pattern (directivity), polarization, and impedance bandwidth [53]. Other design constraints, such as antenna size and sensitivity, must be considered. The antenna’s sensitivity reflects its ability to harvest energy and operate at low input power levels. The receiving antenna should be low profile, given the low size of the sensors. A review of commonly considered minimization techniques is provided in [54].

1. Receiving antenna operating frequency

The antenna’s operating frequency depends on the transmitted signal’s frequency or those available at the sensor node’s location. In this case, a distinction will be made between multi-band or wideband antennas and single-band antennas. Multiband and wideband antennas are suitable for harvesting ambient energy, while single-band antennas are suitable when a dedicated source (WPT) is used to power the rectenna.

2. Receiving antenna gain

Regardless of the rectenna feeding technique, high gain increases the amount of power picked up by the antenna. A large gain is even more important in the case of the WPT to compensate for the path losses. However, in ambient energy harvesting solutions, a moderate gain is sufficient since the increase in gain is very often related to the rise in the antenna size, as expressed below [55].

(9) $$G_{R}=\frac{4\pi A_{e}f}{c}$$

where $G_{R}$ is the receiving antenna gain, $A_{e}$ is the antenna’s effective area, c is the speed of light, and f is the operating frequency.

3. Receiving antenna radiation pattern

The radiation pattern plays a vital role in the amount of harvestable energy. We will distinguish directional diagrams from omnidirectional diagrams. In ambient energy harvesting, given the unknown direction of the incident waves, omnidirectional patterns are more appropriate. Strongly directive patterns are adequate in the case of the WPT.

4. Receiving antenna polarization

Polarization reflects the direction of the waves received by the antenna. The power received by the antenna depends on the polarization efficiency, which is defined as the ratio between the polarization of the transmitting antenna and that of the receiving antenna. An antenna has three main types of polarization: linear, circular, and elliptical. In energy harvesting applications, circular polarization is preferable because it maintains a constant output voltage despite the rotation of the transmitting antenna or rectenna [56].

5. Receiving antenna bandwidth and size

Generally, the broadest possible frequency band is preferable to simultaneously harvest energy from several sources. The bandwidth of an antenna is defined as the frequency deviation for which the reflection coefficient is less than a given value, generally −10 dB. The reflection coefficient denoted by Γ is defined as follows [57]:

(10) $$\Gamma =\frac{Z_{a}-Z_{s}}{Z_{a}+Z_{s}}$$

where $Z_{a}$ is the antenna impedance and $Z_{s}$ is the source impedance.

The bandwidth BW of an antenna is related to its quality factor Q and its resonant frequency $f_{R}$ as follows:

(11) $$\mathrm{BW}=\frac{f_{R}}{Q}$$

Given the miniature size of the WS (a few ${\mathrm{mm}}^{3}$ in volume [58]), the receiving antennas must be compact and embeddable. A reduction in the size of the antennas will lead to a reduction in the quality factor, thus giving rise to a broader bandwidth. However, this reduction will affect the antenna’s gain, as shown in Equation (9). The fundamental boundary between antenna size and efficiency was defined by Wheeler et al. in 1947 [59], and it states that for a wave emitted at frequency f, the maximum dimension of the electrically small antenna is less than $\frac{1}{k}=c/2\pi f$ and enclosed in a sphere of radius a with ka<1; where k is the wave vector. In this case, the minimum quality factor allowing low losses is defined as follows:

(12) $$Q\geq \frac{1}{k^{3}a^{3}}+\frac{1}{ka}$$

#### 4.1.2. Leading Solutions Commonly Used to Achieve Usable Power Levels

To capture the most power while maintaining acceptable dimensions, a compromise must be made between the different characteristics of the receiving antenna. The conversion efficiency $\eta_{\mathrm{ant}}$ of the antenna is evaluated, considering losses in dielectrics, conductors, and other materials as follows [54]:

(13) $$\eta_{\mathrm{ant}}=\frac{\left(1-\Gamma^{2}\right)R_{r}}{R_{r}+R_{m}}$$

where Γ is the reflection coefficient, $R_{r}$ is the radiation resistance, and $R_{m}$ the resistance of material loss in the antenna. 

The design method to optimize the antenna conversion efficiency while maintaining a reasonable volume uses high dielectric constant and low-loss materials [13]. Several types of antennas, including dipole, patch, fractal, and spiral antennas, have been proposed for RF-EH. Among these, patch antennas, due to their lightweight, low cost, and ease of integration, are the most considered [55]. However, its low bandwidth, volume constraints, and need for flexibility have led to a preference for dielectric resonator antennas (DRA). The main characteristics of dielectric resonators are their high permittivity, low dielectric losses, and thermal stability around the resonance frequency. These antennas also offer the advantage of reconfiguring their radiation pattern according to exciting modes. In [60], for example, a dielectric resonator antenna is designed to harvest energy in a wide frequency band ranging from 1.67 to 6.7 GHz while offering a gain of 8.7 dBi. At 1.8 GHz, maximum conversion efficiency of 61.4% is achieved, giving an output voltage of only 400 mV. Recall that the power supplied by the antenna must be shaped by the RF/DC converter, and the latter can only operate at a specific power threshold. To achieve usable power levels in RF-EH applications, some of the leading solutions proposed are multi-band, reconfigurable antennas, and antenna arrays.

1. Multi-band antenna

Multi-band antennas are more considered for harvesting the ambient RF energy available due to the operation of telecommunications equipment. Harvesting RF energy in several frequency bands makes it possible to increase the captured power as follows [54]:

(14) $$P_{R}=\overset{n}{\underset{i=1}{\sum }}P_{f_{i}}$$

where $P_{R}$ is the total power received by the antenna, n is the number of frequencies, and $P_{f_{i}}$ is the power received at the ith frequency.

Multiband antennas are often obtained by considering the pi-shaped radiating elements [61]. The performances offered by such antennas vary considerably from one frequency band to another. For example, in [62], a triple band antenna (900 MHz, 2100 MHz, and 2.36 GHz) using a Rogers RO4350 patch is proposed. The highest gain value obtained is 2.64 dBi at 2.025 GHz. The maximum conversion efficiency is 61.3%, achieved at the frequency of 1.575 GHz. Multiband antennas with gain enhancement are also obtained using DRAs. For example, a rectenna based on a semi-cylindrical dielectric resonator antenna is proposed for WSs dedicated to smart cities in [56]. The proposed design offers a minimum gain of 4.5 dBi. It allows efficient harvesting of RF energy in the 5.725–5.875 GHz and 5.925–7.125 GHz frequency bands with corresponding conversion efficiencies of 66.6% and 65.2% achieved at 11 dBm input power. To further improve this performance, reconfigurable antennas are also offered.

2. Reconfigurable antenna

The reconfigurability of the antenna consists of modifying its radiation properties, which is achieved by changing the structure of the antenna. In this way, the trade-off between bandwidth and antenna size is realized since only one antenna is used. In general, the reconfigurability of the antenna is achieved by slots on the surface of the radiating element and connecting these surfaces by switches. In the specific case of patch antennas, the switches are made using PIN diodes [63]. In the ON state, the PIN diode has a resistive behavior, while it behaves like a capacitance in its OFF state. This is how the current distribution in the patch and resonant frequency varies. In [64], for example, a compact rectenna is proposed operating at 5.2 and 5.8 GHz with respective conversion efficiencies of 70.5% and 69.4%. The switches are made using BAP51-02-type PIN diodes. As seen above, in most cases, reconfigurability is usually achieved on only two frequency bands. However, ambient RF sources exist in more than two frequency bands depending on the sensor’s location.

3. Array antennas

To overcome the limitations of a single receiving antenna, antenna arrays are considered for RF energy harvesting. The total power of an antenna array is obtained by combining the output power of two or more antenna elements. For example, in [28], Manuel et al. propose ambient harvesting energy in four frequency bands (DTV, GSM900, GSM1800, and 3G) with four different rectennas. First, the outputs of the rectennas are connected in series, and the same circuit is used to shape the energy before storage. One of the problems posed by this architecture is that the rectennas connected in series are crossed by the same current, which prevents each rectenna from operating at its optimum power point. The harvested energy is individually processed for each rectenna [28]. The topology used is parallel, and each rectenna can operate at its optimum power point. However, this is done at the expense of clutter because four different circuits are used to manage the energy supplied by each rectenna. Thereby, although the antenna array solution improves the amount of harvestable power, it still requires the design of additional circuits to process signals from several sources [54].

The previous summarizes the requirements of receiving antennas, and the following subsection deals with RF/DC conversion.

### 4.2. The RF/DC Converter

Its role is to transform the RF energy captured by the antenna into DC energy suitable for powering the WSs. For this, Schottky diodes or complementary metal oxide semiconductor (CMOS) technology can be used. CMOS technology is more sensitive than Schottky diodes; however, their leakage current is very high, which causes power loss and degrades the overall system efficiency [12]. For this reason, we will only deal in this section with the design requirements of rectifiers based on Schottky diodes. Overall, the performance achieved by the RF/DC converter will depend on the type of diodes used and topology of the rectifier.

#### 4.2.1. Main features of Schottky Diodes for RF Energy Harvesting

**Figure 6:**
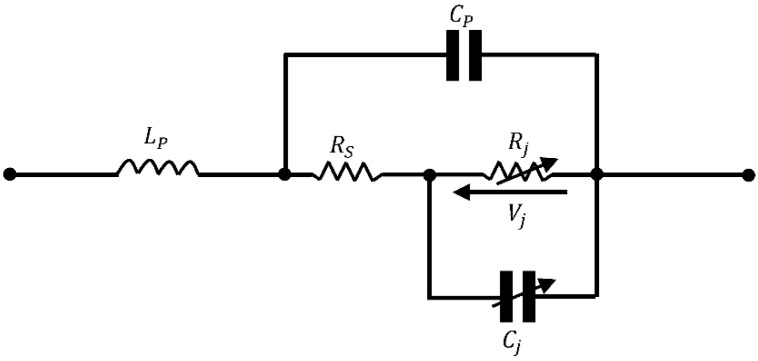
Small signal model of Schottky diode.

Unlike standard diodes such as the 1N400X series, Schottky diodes offer high switching speed, low forward voltage drop, low power consumption, and low parasitic effects. For these reasons and given the high frequencies of radio frequency waves, these diodes are preferred in the design of rectennas. The performance of the RF/DC converter varies depending on the used Schottky diode and operating frequency of the rectenna. For circuit analysis, the low-frequency model of the Schottky diode is established in [65] and shown in Figure 6. Commonly used Schottky diodes and their parameters are provided in Table 3 where $V_{B}$ represents the breakdown voltage. The main manufacturers are Skyworks, Avago, and Macon [55].

As shown in Figure 6, the diode consists of series resistance, $R_{s}$, which dissipates all the RF energy that travels through it in heat. This resistance’s high value then degrades the RF/DC efficiency. It represents the equivalent resistance from the combination of the bulk layer of the silicon substrate, bond wire, lead frame, etc.

$R_{j}$ in Figure 6 is the video resistance, depending on the current flowing through the diode. It is shown in [24] that an increase in the value of this resistance (beyond 4 kΩ) will increase the value of the reflection coefficient as defined in Equation (10). The voltage $V_{j}$ represents the voltage across the semiconductor-metal junction. $L_{P}$ and $C_{P}$ exhibit parasitic inductance and parasitic capacitance, respectively. Parasitics are mechanical and electrical unwanted characteristics that limit the circuit’s performance. $L_{P}$ is the inductance associated with the external terminations of metal that connect the internal component with the external circuit. The capacitance $C_{P}$ is placed in parallel with the diode since all packages of solid material have dielectric constants related to capacitors. $C_{j}$ in Figure 6 is a nonlinear junction capacitance; its value is affected by the thickness of the epitaxial layer and diameter of the Schottky diode. Varying $C_{j}$ shifts the tuned frequency position, thus causing a mismatch in the resonant frequency [24]. The dependence of $C_{j}$ on the output voltage of the RF/DC converter is expressed in [66] as follows:

(15) $$C_{j}=C_{j_{0}}\sqrt{\frac{V_{j}}{V_{j}+V_{DC}}}$$

where $C_{j_{0}}$ is the diode’s zero bias junction capacitance and $V_{DC}$ the voltage across the load resistance.

The conversion efficiency of a Schottky diode is closely related to its internal electrical characteristics, operating frequency, and incident RF power level. Considering a diode arranged in parallel with the receiving antenna, the RF/DC conversion efficiency was defined in [66] as follows:

(16) $$\eta_{RF/DC}=\frac{1}{1+A+B+C}$$

where $\eta_{RF/DC}$ is the RF/DC conversion efficiency with A,B, and C defined as below.

(17) $$\{\begin{array}{l} A=\frac{R_{L}}{\pi R_{S}}{\left(1+\frac{V_{j}}{V_{\mathrm{DC}}}\right)}^{2}\left[\theta_{\mathrm{on}}\left(1+\frac{1}{2\;\cos^{2}\theta_{\mathrm{on}}}\right)-1,5\tan\theta_{\mathrm{on}}\right]\\ B=\frac{R_{S}.R_{L}.C_{j}^{2}.\omega^{2}}{2\pi }\left(1+\frac{V_{j}}{V_{\mathrm{DC}}}\right)\left[\frac{\pi -\theta_{\mathrm{on}}}{\cos^{2}\theta_{\mathrm{on}}}+\tan\theta_{\mathrm{on}}\right]\;\\ C=\frac{R_{L}}{\pi R_{S}}\left(1+\frac{V_{j}}{V_{\mathrm{DC}}}\right)\frac{V_{j}}{V_{\mathrm{DC}}}\left[\tan\theta_{\mathrm{on}}-\theta_{\mathrm{on}}\right] \end{array}$$

where $R_{L}$ is the load resistance, and $V_{DC}$ is the voltage across the load resistance; the other parameters have been defined above. $\theta_{\mathrm{on}}$ is the forward-bias turn-angle diode. It is a dynamic variable depending on the input power of the diode; it is defined in [66] as follows:

(18) $$\tan\theta_{\mathrm{on}}-\theta_{\mathrm{on}}=\frac{\pi R_{S}}{R_{L}\left(1+\frac{V_{j}}{V_{\mathrm{DC}}}\right)}$$

**Figure 7:**
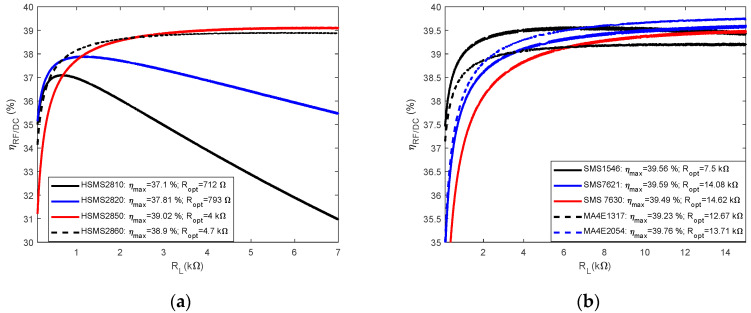
Optimal loads of commonly used diodes. (**a**) Avago diodes. (**b**) Skyworks and Macon diodes.

Considering Equations (16)–(18), as well as the electrical characteristics of the diodes (Cf. Table 3), a comparison of the conversion efficiency as a function of the load resistance of the commonly considered diodes is shown in Figure 7. The analyses were carried out with MATLAB software. The operating frequency is 900 MHz, and the desired DC voltage is fixed at 2.7 V. The maximum conversion efficiency and optimum load resistance are captioned on each figure. Overall, to achieve the desired performance in terms of DC voltage, the efficiency levels vary between 30% and 40%. Figure 7a shows the performance achieved with Avago diodes, and the desired performance is achieved at lower load resistances. The HSMS 2850 diode offers the best performance. In Figure 7b, the results achieved with the Skyworks and Macon diodes are shown. Performances are slightly higher than those achieved with Avago diodes but at the expense of much higher optimal resistances.

**Figure 8:**
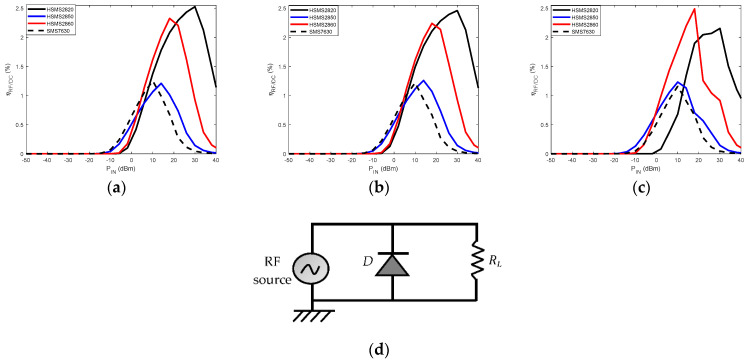
Comparison of the detection threshold of the diodes. (**a**) 900 MHz GSM band. (**b**). 2.45 GHz ISM band. (**c**) 5.8 GHz ISM band. (**d**). Schematic for the diode detection threshold.

The Schottky diode detection threshold is a critical selection criterion depending on the harvestable power level. Figure 8 illustrates the performance achieved with four of the diodes whose characteristics are given in Table 3. The RF/DC conversion efficiency is analyzed according to the input RF power level. The analyses are carried out at 900 MHz, 2.45 GHz, Industrial Scientific and Medical (ISM) band, and 5.8 GHz ISM band with the Advanced Design Software (ADS). The simulated circuit to achieve these performances is the same, having been considered in [67]. The simulated circuit is shown in Figure 8d; and for each of the diodes, the load resistance is set to its optimum value labeled in Figure 7.

In Figure 8, low RF/DC conversion efficiency levels are observed, which are caused by reflection losses due to the lack of the matching filter. In Figure 8a,b, for the 900 MHz and 2.45 GHz frequency bands, the SMS 7630 diode is the most sensitive for detecting input power levels below −10 dBm. For these two frequency bands, the HSMS 2860 diode is the most appropriate in the case of wireless power transfer, where power levels of 20 dBm can be emitted. At 5.8 GHz in Figure 8c, the HSMS 2850 diode is the most sensitive, and beyond −5 dBm, the HSMS 2860 diode is more appropriate for the design.

The findings in this subsection illustrate the sensitivity of the RF/DC converter to the internal electrical characteristics of the rectifying diode. In the following subsection, the impact of rectifier topology is discussed.

#### 4.2.2. Main Rectifier Topologies

The topology determines the arrangement of the diode(s) in the rectifier circuit. The main topologies are half-wave rectifiers, full-wave rectifiers, and voltage multipliers [12].

1. Half-wave rectifiers

**Figure 9:**
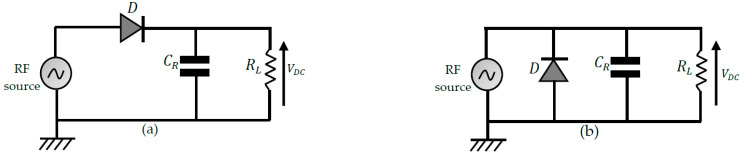
Half-wave rectifier topologies. (**a**) SSD. (**b**). SPD.

These topologies use a single diode allowing only one alternation of the RF wave to be transferred to the load. Two configurations shown in Figure 9 can be used: a single series diode (SSD) and a single parallel diode (SPD). Capacitor $C_{R}$ is used to filter the rectified voltage. Half-wave topologies using few components have the advantage of presenting fewer losses than full-wave topologies. They are, therefore, more suitable for harvesting energy at very low input power levels. However, it remains less efficient for usable power levels than full-wave topologies. For example, in [68], a reconfigurable rectenna is proposed depending on the RF input power level. The SSD topology demonstrates a maximum conversion efficiency of less than 50%, unlike a full-wave topology, which achieves nearly 80% maximum efficiency. This is one of the main reasons why full-wave topologies are considered more in the design of rectennas.

2. Full-wave rectifier topologies

**Figure 10:**
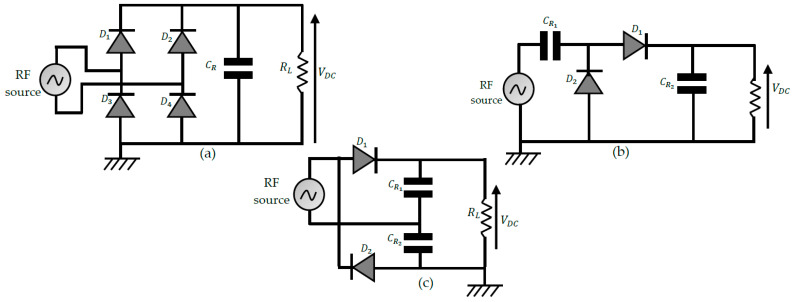
Full-wave rectifier topologies. (**a**) FB. (**b**). SVD. (**c**) LVD.

These topologies transfer much power to the load as half-wave topologies. A distinction is made between the full-bridge (FB) rectifier and the voltage doubler rectifiers. The different configurations are shown in Figure 10. Voltage doublers (VD) use two filter capacitors and provide a DC output voltage twice that of FB rectifiers. VDs are of two types: Schenkel VD (SVD) and Latour VD (LVD). The performances achieved in output DC voltage are almost similar for the two voltage doublers. However, it is shown in [69] that SVD exhibits more matching losses than LVD, making the LVD more suitable for designing miniature rectennas that do not require a matching filter.

In general, the choice of rectifier topology depends on two factors, the conversion efficiency η, and DC output voltage $V_{DC}$. These two parameters are related to the RF input power, $P_{IN}$ and load resistance $R_{L}$ as follows:

(19) $$\eta \left(\%\right)=100\times \frac{V_{DC}^{2}}{R_{L}.P_{IN}}$$

**Figure 11:**
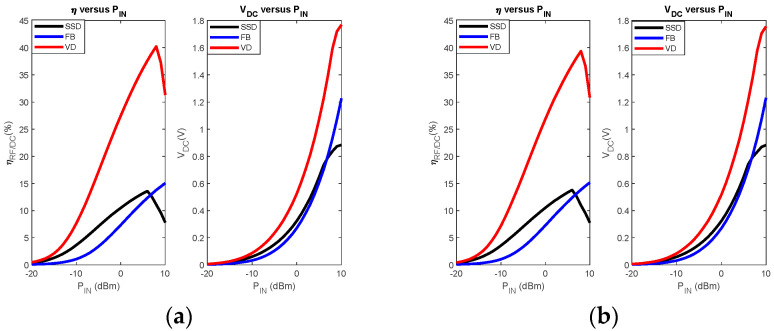
Performance comparison of main commonly used rectifier topologies. (**a**) 900 MHz GSM band. (**b**). 2.45 GHz ISM band.

For filtering capacitance fixed at 3.3 pF, a comparison of the performances offered by the three topologies of rectifiers (SSD, FB, and VD) based on the HSMS 2850 Schottky diode is shown in Figure 11. The ADS software is used for the simulations.

The results below show that the VD topology is the one that offers the best performance over the considered input power range. The results also show that below 5 dBm, the SSD topology is more efficient than an FB rectifier. This performance comparison was used in [68] in the design of a rectenna reconfigurable by the rectifier type. The proposed rectenna operated at −5 dBm input power with the SSD topology, while the FB topology was selected to harvest energy at 25 dBm input power. The results of Figure 11 justify that VD rectifiers remain the most considered for the design of rectennas. Additionally, they offer the possibility of further amplifying the DC output voltage of the rectifier by configurations known as multi-stage voltage doublers.

3. Multistage voltage doublers (MSVD) rectifiers

**Figure 12:**
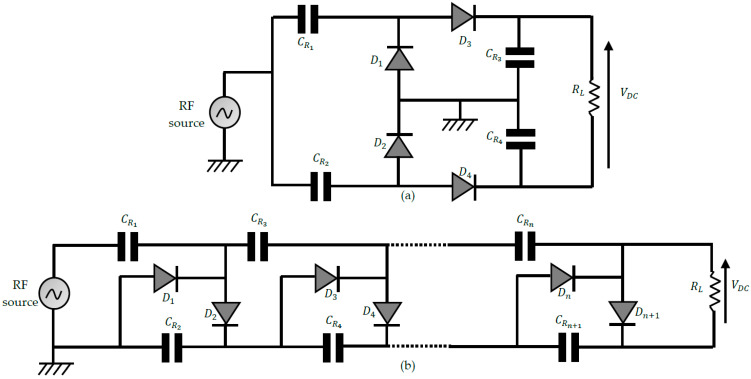
Voltages multiplier topologies. (**a**) Greinacher rectifier configuration. (**b**). Cockcroft-Walton voltage multiplier.

The performance of rectennas in DC output voltage is further improved using voltage multiplier rectifiers. The main configurations reported are shown in Figure 12. In Figure 12a, the Greinacher rectifier [70] is displayed; it is a two-stage voltage multiplier arranged in a bridge configuration. The diodes are positioned so that the bias voltage of each is generated by the output of the previous diode, thus reducing the need for an external power supply. Figure 12b shows a Cockcroft-Walton n-stage voltage multiplier rectifier, also known as the Villard voltage multiplier [71]. The output DC voltage of a voltage multiplier is linked to the number of stages n and the input voltage level $V_{in}$ as follows:

(20) $$V_{DC}{}_{OC}=2n\left(V_{in}-V_{th}\right)$$

where $V_{DC}{}_{OC}$ is the open circuit voltage and $V_{th}$ is the forward voltage drop.

**Figure 13:**
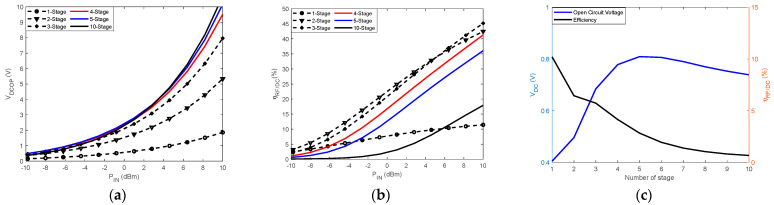
Performance comparison of multistage rectifiers. (**a**) Voltage. (**b**) Efficiency. (**c**) The trade-off between voltage and efficiency.

Voltage multipliers are known to increase the output voltage of the rectenna. However, the number n of stages is a critical design parameter when analyzing the conversion efficiency [72]. Figure 13 shows the results of the analysis of a voltage multiplier with up to 10 stages. ADS software is used for the simulations, and the analyses are based on the characteristics of the Schottky diode HSMS 2850 from Avago.

As shown in Figure 13a, increasing the number of stages automatically increases the DC output voltage. We also note that beyond four stages, the improvements are no longer significant. However, Figure 13b shows degradation of the RF/DC conversion efficiency with the increase in the number of stages in the circuit. In Figure 13c, the rectenna is fed with an RF power of −8 dBm, and the number n of stages is varied to obtain the conversion efficiency and open-circuit DC voltage each time. The results reveal that the compromise is reached by using 3-stage voltage multipliers for this level of input RF power.

### 4.3. The Matching Filter

#### 4.3.1. General Principle and Main Features

**Figure 14:**
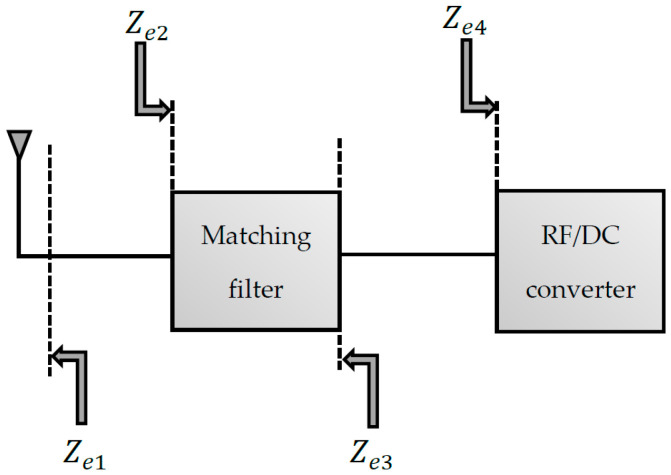
The principle of the impedance matching.

The diodes used to carry out the RF/DC conversion have a non-linear behavior, thus generating harmonics of the operating frequency. These re-radiated harmonics interfere with the fundamental frequency’s waves, giving rise to degradation of the overall performance of the rectenna. The matching filter’s role is to block the harmonics generated by the rectifier circuits. The principle of matching is shown in Figure 14, and mathematically, it is achieved when:

(21) $$\{\begin{array}{c} Z_{e1}=Z_{e2}^{*}\\ Z_{e3}=Z_{e4}^{*} \end{array}$$

where $Z_{e1}$ is the output impedance of the antenna (generally 50 Ω), $Z_{e2}$ is the input impedance of the matching filter seen from the antenna, $Z_{e3}$ is the output impedance of the matching filter seen by the rectifier circuit, and $Z_{e4}$ is the input impedance of the rectifier circuit seen by the matching filter. $Z_{e2}^{*}$ and $Z_{e4}^{*}$ represent the conjugate complexes of $Z_{e2}$ and $Z_{e4}$, respectively. For rectenna operability, the matching filter must be able to match the antenna and rectifier circuit for any level of input power and operating frequency. Thus, the filter must have a small form factor and a low-quality factor.

#### 4.3.2. Main Impedance Matching Techniques

**Figure 15:**
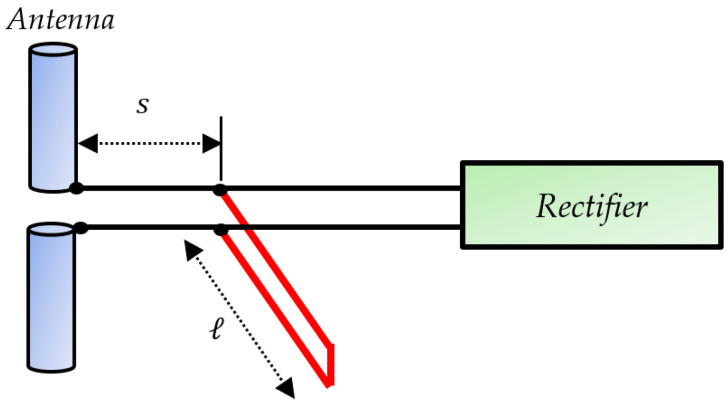
Shunt stub matching technique.

Many techniques exist to realize the principle shown in Figure 14. An ideal method uses a shunt stub [57], as shown in Figure 15. In this method, the length s is varied to match the real part of the antenna to that of the rectifier circuit. The variation of the length ℓ of the section in shunt makes it possible to adjust the susceptance of the section to equal it in amplitude but in phase opposition to the susceptance from the connection point. The primary electrical components, R, L, and C are also considered to design the matching networks. The resistance translates the real part of the impedance, and the reactive components L and C are used for the imaginary part.

**Figure 16:**
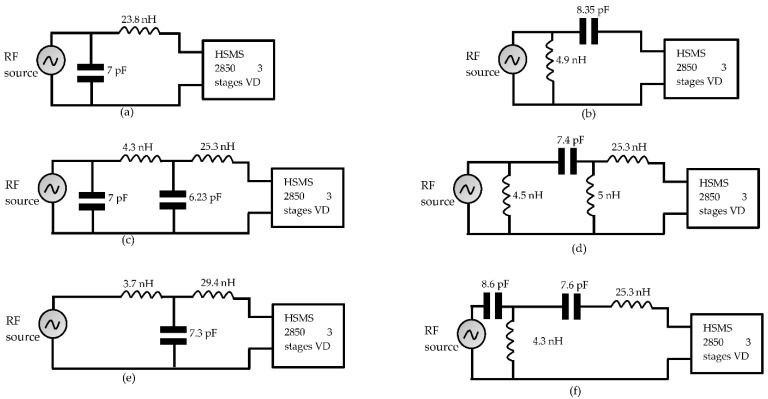
Designed matching filters for 3-stages voltage multiplier. (**a**) L low pass. (**b**) L high pass. (**c**) π low pass. (**d**) π high pass. (**e**) Tee low pass. (**f**) Tee high pass.

Generally, the use of resistors in matching filters generates losses in the circuit; thus, only the imaginary part of the impedance is considered in the adaptation [73]. There are two main matching filters for rectennas: the transformer coupling [74] and the LC network [73]. The various commonly used topologies are shown in Figure 16. This figure shows that LC filters can be L-, π-, and Tee-type. A comparison of the characteristics and performances achieved with these different filter configurations has been proposed in [73,75]. In [73], these characteristics have been defined by considering a voltage doubler rectifier and several multistage voltage doublers with 3, 5, and 7 stages. The HSMS 2852 diode was considered for the design. Overall, it is obtained that L-type filters offer better conversion efficiency than π-type filters. However, pi-type filters allow for greater bandwidth. Tee-type filters offer much better DC output voltage levels than the previous two configurations. The same previous findings were observed in [75] for the design of a 900 MHz rectenna based on a 3-stage voltage multiplier rectifier with HSMS 285B diode.

**Figure 17:**
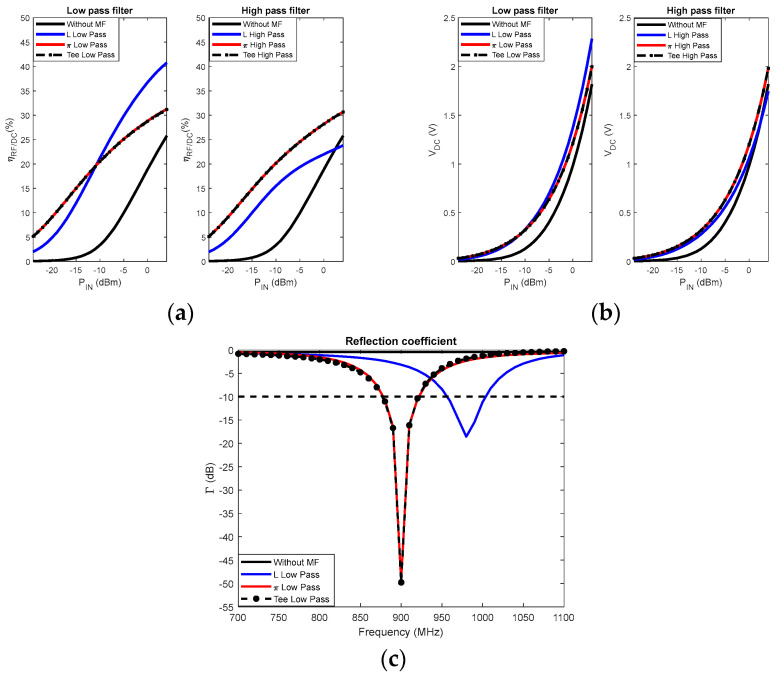
Comparison of performance achieved with various filter configurations. (**a**). Efficiency. (**b**) Voltage. (**c**) Reflection coefficient.

The choice of filter component values according to the used filter topology and the characteristics in terms of bandwidth (quality factor) can be made analytically based on the principle shown in Figure 14. Yet, most designers use ADS software to size the matching filter. This software integrates tools allowing the adjustment of the components of the filter to ensure a good matching between the antenna and the rectifier. The ADS Matching utility tool determines the values of the L and C components according to the chosen filter configuration. Other optimization tools are integrated into the software and will allow the refinement of the values of the components to achieve the specific objectives defined by the user. The simultaneously considered goals are the maximization of the conversion efficiency and the DC voltage combined with the minimization of the reflection coefficient [67]. The optimization techniques used in the software are mainly based on the gradient method and that of Newton. All these design steps are presented in [55]. Applying the steps mentioned in this previous study, to a 3-stage voltage-multiplier rectifier, the schematics obtained according to the filter configuration are shown in Figure 16. The analyses are based on the characteristics of the Schottky HSMS 2850 diode, and the load resistance is 5 kΩ, corresponding to the optimum load of the 3-stage voltage doubler rectifier. A comparison of the simulated (with ADS software) performance achieved with varying configurations of the filter is shown in Figure 17.

Overall, Figure 17 shows that the performances achieved are almost similar for the π-type filter topologies and the Tee-type filters. The reflection coefficients shown in Figure 17c are better for these two topologies. However, for an L-Low pass filter, the best performances in terms of DC voltage and conversion efficiency are better under these operating conditions. Again, as with the antennas and rectifiers, a trade-off must be made between filter bandwidth and conversion efficiency. All the above summarizes the design strategies of the matching filters, the sensitivity of the different configurations presented to the input RF power level, and the internal electrical characteristics of the diodes used.

### 4.4. DC/DC Converter

#### 4.4.1. Brief Description

Typically, the voltage or power generated by the rectifier may be low to power the WS or storage device. The DC/DC converter thus makes it possible to adapt the voltage to the load. For this, it must be preceded by a controller making it possible to ensure the maximum power point tracking (MPPT) given that the conversion efficiency of the rectifier depends on the load resistance. The MPPT aims to obtain the maximum power by adjusting the current or the voltage according to the current-voltage curve of the rectenna.

**Figure 18:**
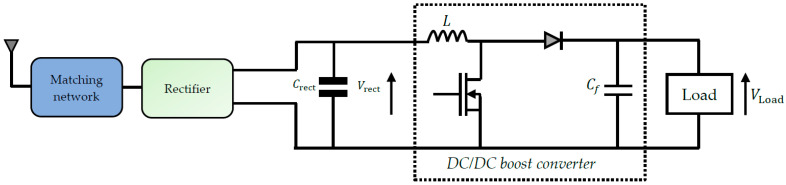
Structure of DC/DC boost converter.

There are several DC/DC converters; the two main structures are the DC/DC buck converter and the DC/DC boost converter. The choice of the converter depends on the voltage values $V_{\mathrm{rect}}$ of the rectifier and the supply voltage of the used storage device. When harvesting RF energy, the boost converter shown in Figure 18 is commonly used [76]. A PMOS transistor switches and inductor L to provide a stable and regulated output DC voltage. The gate control signal of the PMOS transistor is a square wave generated by an external oscillator. When a low voltage $V_{\mathrm{rect}}$ is present at the converter’s input, it delivers a voltage $V_{\mathrm{Load}}$. Feedback of this voltage makes it possible to power the oscillator, which thus generates the control signal.

#### 4.4.2. Main Features and Techniques for Optimizing DC/DC Conversion Efficiency

The brief description of the operation of the DC/DC converter given above shows that the complexity in the design resides at the level of the control circuit of the PMOS transistor. This circuit generally integrates other NMOS transistor-based switches as well as signal comparators. One factor affecting the rectenna’s overall conversion efficiency is the power loss in the oscillator circuit. Most designs use two oscillators: one at low frequency and the other at high frequency [77]. This increase in the number of components then increases losses in the circuits. One of the proposed solutions to overcome this drawback is using a single externally biased comparator-based oscillator circuit as an astable multivibrator. In [24], for example, the comparator is realized using the LTC1540 nano-power detector with a switching inductance of 330 µH. The circuit made it possible to boost the voltage from 0.5 to 2.25 V.

Another technique for transferring the maximum power ensures that the boost converter’s input impedance must emulate the optimum rectenna load. Generally, the emulation resistor $R_{\mathrm{em}}$ is defined as follows [24,77]:

(22) $$R_{\mathrm{em}}=\frac{2L}{ft_{1}^{2}k}\left(1-\frac{V_{\mathrm{rect}}}{V_{\mathrm{Load}}}\right)$$

where L is the inductance, f is the frequency of the external oscillator, $t_{1}$ is the switch ON time of the control circuit, k is a low-frequency pulse duty cycle when the boost converter operates in pulsed mode, $V_{\mathrm{rect}}$ is the output voltage of the rectifier, and $V_{\mathrm{Load}}$ is the voltage delivered to the load. Using the technique of emulation resistance in [77], conversion efficiency of almost 90% is achieved at 700 µW of incident power with a switching inductance set at 220 µH.

Additionally, to resistance emulation, an alternative technique used to optimize the performance of boost converters is known as particle swarm optimization. The particle swarm optimization technique aims to find the best values of inductor and on-time, which provide the maximum efficiency. By combining this technique with the emulation resistor in [78], the conversion efficiency achieved was 9.25% higher than that obtained without the DC/DC converter. In [79], the implementation of the converter achieves 87.7% efficiency at only −30 dBm. All the above justify the need and design considerations of DC/DC converters in the realization of rectenna.

### 4.5. Storage Element 

When the energy harvested by the rectenna is insufficient to power the WS directly, it must be stored. The performances of storage devices are compared using the Ragone diagram [80]. This diagram represents the energy density in Wh/kg as a function of the power density in W/kg. The storage element can be a simple capacitor, a supercapacitor, or a battery. Compared to the other two storage devices, capacitors are inexpensive; however, they remain very little used in the design of rectennas due to their low power density.

Supercapacitors have a higher energy density than capacitors and a higher power density than batteries. It has been shown in [81] that they have a longer life in charge and discharge cycle and a lower internal impedance. For example, in [82], the internal impedance of a 350 F supercapacitor is of the order of milli-ohms. This low internal impedance results in higher charging efficiency than the other two storage devices. The major disadvantage of supercapacitors is their high self-discharges, thus degrading the overall efficiency of the rectenna.

Compared to supercapacitors, batteries offer better energy density at the expense of a short lifetime and low power density. A compromise was achieved previously by introducing a new component known as the supercapattery. The electrode in supercapatteries combines the most effective components in supercapacitors and batteries, carbon nanotubes, and redox materials [83]. A supercapattery has a higher energy storage capacity than a supercapacitor and a faster charge and discharge value than a battery.

This section of the article outlines design considerations for a rectenna and reviews the various techniques for achieving usable power levels. Looking also at the findings of the previous section (dealing with rectenna feeding techniques), the amount of power available for the WS remains relatively low. The following section then deals with commonly considered solutions to minimize the energy consumption of WSs.

## 5. Minimization of the Energy Budget of the WS (MEB-WS)

The possibility of considering autonomous WSs based on RF-EH is mainly also due to the efforts made in recent years to minimize the energy budget of the WSs. These efforts are commonly known as WS lifetime extension [84]. Three research axes are proposed in the literature to extend WS lifetime or minimize WS spent energy regarding the choice of energy conservation hardware for sensors, network topology selection, and communication protocol adoption.

### 5.1. Choice of Hardware Components for Minimizing Ws Energy Consumption

With MEMS technology, it is now possible to have small, low-power, and low-cost marketed WSs. The first objective targeted by MEMS technology is the miniaturization of the sensing unit, which implies a need to have a miniature rectenna. To extend the lifetime of WSs, MEMS technology also aims to reduce more and more the consumption of the different components of the WS [58]. Table 4, Table 5 and Table 6 summarize some energy consumption levels of commonly used components regarding sensing, processing, and communication units, respectively [85]. Table 4 shows that the current consumption of the sensor varies hugely according to the type of sensor. Another parameter to be considered is the measurement range, which can also be obtained from the component datasheet.

Table 5 shows that the Texas Instruments MSP430 series microcontrollers offer the lowest consumption levels. In [34], for example, the features of these microcontrollers were considered to demonstrate that ambient RF power levels are enough to support the activation of battery-less WSs for IoT applications. For this purpose, the authors designed a reconfigurable rectenna to be able to harvest ambient RF energy over several frequency bands. Table 6 shows that the communication unit is the biggest consumer of energy. These consumption levels may vary according to the used throughput, usually too low, and the power level used by the transmitter to reach some communication ranges.

Based on the characteristics described in Table 4, Table 5 and Table 6, and depending on the application, a combination of the various components can be performed to minimize the overall consumption of the WS. Most applications where the physical phenomenon varies very slowly, such as temperature variations, are less energy-intensive due to thermal inertia. In such applications, it is not necessary, for example, to perform measurements every second. In the following, we present the influence of the network topology on WS consumption.

### 5.2. Influence of the Network Topology on WS’s Energy Consumption

**Figure 19:**
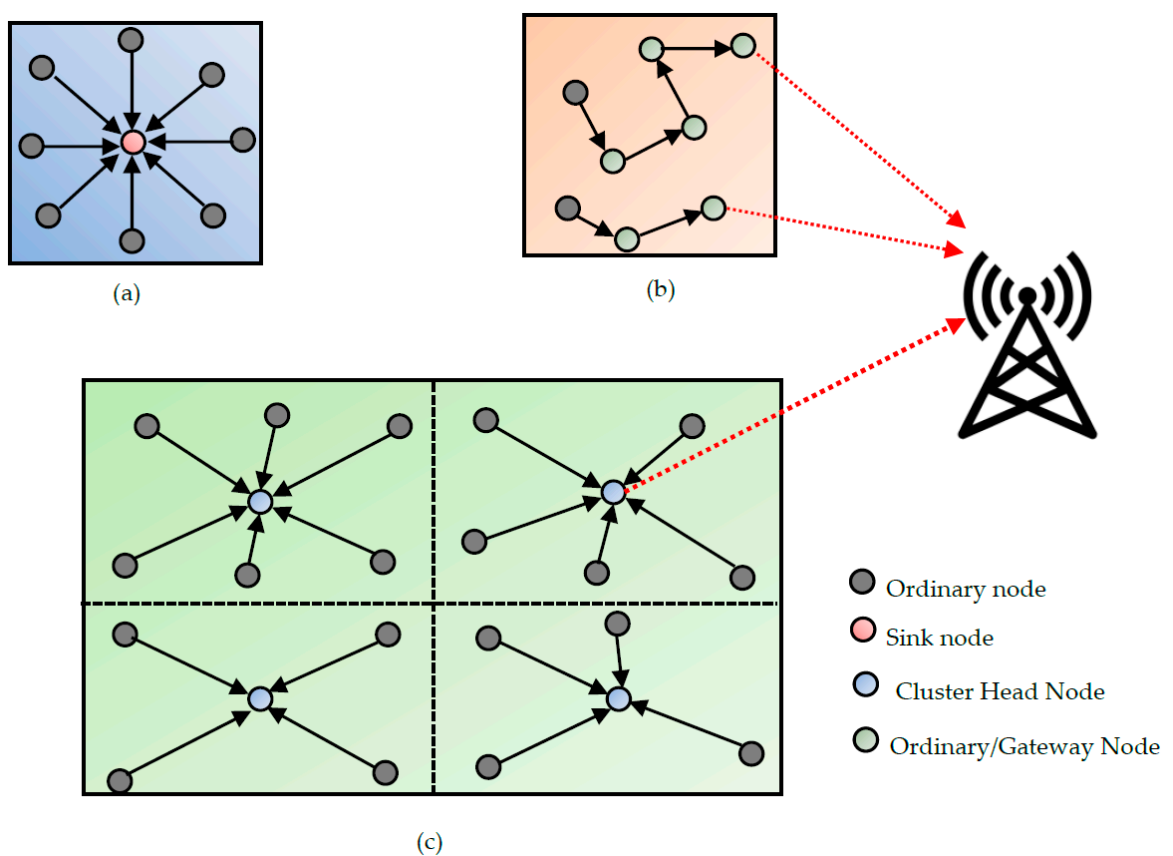
Main network topologies. (**a**) Star, (**b**) Mesh, (**c**) Cluster.

The topology determines the organization of WSs in the network. Generally, the selected topology depends on the communication protocol implemented inside the WS. Depending on the communications standards, the topologies that can be supported are detailed in [86]. There are three main topologies represented in Figure 19: star, mesh, and cluster.

The star topology consists of a central node called a coordinator or sink, and several sensor nodes directly transmit their data to the sink. The mesh topology consists of a set of nodes that all have the same function in the network. This is the standard topology of conventional mobile sensor networks. Each WS in the network acts as a relay or gateway for the neighboring node. Finally, the cluster topology is partitioned into WS subgroups called clusters. Each cluster consists of a particular node called the cluster head and other nodes communicating only with their cluster head. These three topologies were compared in [67,87]. It is shown that the star topology is the most efficient regarding energy efficiency; therefore, it is the most widely used topology to support IoT applications [88,89]. In [88], a star network is proposed for IoT applications based on the IEEE 802.15.4e specification using a time-slotted channel hopping protocol. This previous study aims to meet the throughput requirements and extend the WS node’s battery lifetime.

### 5.3. The Main Communication Protocols in RF-EH-WSs

In several studies, the communication module is considered the largest consumer of energy [85]. Therefore the research is more focused on the link layer, which deals with access methods to the communication channel [90]. The solutions considered are medium access control (MAC) protocols that reduce energy wastage from collisions, overhearing, control overhead, and idle listening [91]. The essential task of a MAC protocol is to arbitrate access to the shared medium to avoid collisions. Other solutions deploying the IoT paradigm on a large scale, thus overcoming the energy costs associated with data transmission, are the low power wide area network (LPWAN) protocols. In this category, the different technologies include long range wide area network (LoRaWAN), narrowband IoT (NB-IoT), and Sigfox [92].

#### 5.3.1. Main MAC Protocols Dedicated to RF-EH-WSs

Transposed to the RF-EH-WS context, the issue of the MAC protocol is how to address both maximizations of harvested energy and minimization of data communication interruption or failure [93]. Noting the low level of the harvested RF energy, most of the current MAC protocols suited for RF energy harvesting introduce adaptive management issues of the duty cycle of sensors. For a battery-powered WS, effective management involves minimizing the energy consumption of a sensor while maintaining the appropriate QoS for exchanged data. In an RF-EH-WS, the management should define a proper time rate to use the harvested energy to allow sensors to operate indefinitely. When this objective is achieved, the sensors and WSN are said to be in the energy-neutral operation (ENO) mode [14]. MAC protocols for RF-EH-WSs come from conventional MAC protocols (battery-powered WSs). The main MAC protocols designed for RF-EH-WSs are discussed in the following subsections.

1. An On-Demand MAC (ODMAC) protocol

The purpose of this protocol is to minimize energy wastage by performing the three following operations: moving the idle-listening time from the receiver to the transmitter, adjusting the duty cycle to maintain the ENO state, and reducing the end-to-end delay by using an opportunistic forwarding scheme [94,95]. In [94], the micro-controllers of the Texas Instruments MSP430 family are used to implement the ODMAC protocol. The results show that the transmitter node can choose its duty ratio, making it possible to improve the throughput. Despite this positive result, in the implementation of this protocol, it is assumed that the energy harvesting process has no impact on the data communication; this is unrealistic and could not be applied in a practical case.

2. A poll-based Medium Access Mechanism (P-MAC)

In this technique, a sink node is used to enable or control the transmission of WSs in the network. Once a sensor node receives the query, it must send its collected data to the sink [96]. The node that sent its data will not be asked at the next poll duration of time because it will be in a charge state. This is known as the charge-and-spend paradigm. [97] Refs. show that the P-MAC protocol is more advantageous than time division multiple access for wireless body area networks. However, as in the case of the ODMAC protocol, it is wrongly assumed that the energy harvesting process has no impact on data communication.

3. Energy Adaptive MAC (EA-MAC) protocol

A WS powered by an RF energy harvesting process is considered a heterogeneous network due to the difference in the energy level of each sensor. The energy harvested by a sensor depends on its deployed position and ambient RF energy found in the surrounding environment. The EA-MAC protocol proposed in [98] takes this feature involving the propagation of the RF signal by offering adaptive management of the sensor’s duty cycle. Considering at the same time the amount of harvested energy and channel concurrence or contention time, the duty cycle should ensure a fair share of the channel between sensors. Although this protocol manages the rate of accumulated energy, it requires a centralized node, generally the sink node, located inside the network, which cannot be fed by the energy harvested concept because of its very high energy requirement.

4. RF-MAC protocol

The RF-MAC protocol is one of the recent optimization solutions dedicated explicitly to RF-EH-WS since it supports many MEB-WS solutions. The RF-MAC protocols are designed to offer solutions for effectively sharing the communication channel by the RF-EH-WSs. This protocol jointly addresses two problems: optimizing the harvested energy and minimizing the interruption to data communication. In [93], the RF-MAC protocol achieves a gain of 300% for throughput and 100% for the harvested energy. These performances are reached by using several RF energy sources to charge the rectenna battery. When designing an RF-MAC protocol, considering several RF sources necessitates keeping and canceling the constructive and destructive interferences, respectively. This protocol uses the same channel for data transmission and energy harvesting. Whenever the received energy level is lower than a pre-set threshold, priority is given to harvesting the energy. One of the key issues in implementing this protocol is time synchronization in the case of high-frequency carrier signals. Another issue is how to quantify the conversion efficiency of the rectenna. In [93,98], the efficiency is evaluated through the battery recharging time. For IoT applications, an RF-MAC protocol addressing how to balance energy efficiency and quality of service was proposed and implemented in [99].

#### 5.3.2. LPWAN Protocols for RF-EH-WS

To reduce the energy cost associated with data transmission, new communication protocols adapted to the needs of IoT applications have been developed in recent years. Among these solutions are the long-range wide area network (LoRaWAN) [100], the narrowband IoT (NB-IoT) [101], and Sigfox [102]. These communication technologies are grouped under the larger group called low-power wide-area networks (LPWAN) and have two objectives, namely energy-efficient communications with a broader range [103]; a review of these different protocols has been proposed in [103,104,105]. LPWANs are classified into two major groups; the first group, LoRaWAN and Sigfox use license-exempt frequency spectra like the ISM bands. The data is transmitted in the licensed frequency bands for the second group comprising the NB-IoT, and the narrowband technology can co-exist in LTE or GSM [105]. The comparative study proposed in [104] has established that Sigfox and LoRa are advantageous in terms of lifetime and capacity, while NB-IoT offers advantages in terms of processing times.

The purpose of this section was to highlight key points aiming to reduce energy consumption in a WS. This consumption is highly influenced by the hardware elements used to build the sensors, network nodes adopted for the organization, and behavior of the retained communication protocol to exchange information within a WS. The communication protocol offers many possibilities in the MEB-WS. In the following section, we will address the issues of the power management module.

## 6. Efficient Management of Harvested Energy: The Power Management Module

Its function is to decide whether to store harvested energy or use it directly for WS activities while optimizing energy efficiency. To achieve this goal, two main problems have been defined, namely: transmission completion time minimization (TCTM) [106,107,108] and short-term throughput maximization (STTM) [109,110]. 

### 6.1. Transmission Completion Time Minimization (TCTM) Problem

**Figure 20:**
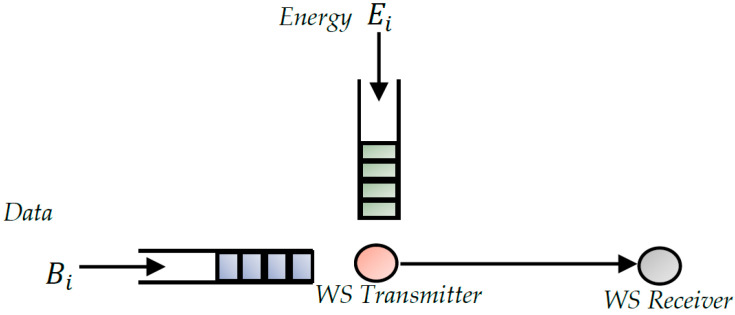
Energy harvesting communication model for solving TCTM problem.

Given that some data bits are transmitted while energy is harvested, the idea is to minimize the duration in which all transmitted bits reach the receiving WS or base station. The basic model to illustrate this problem is shown in Figure 20. This model independently considers the harvested energy and data transmission rate [106]. Recall that it is difficult to accurately predict the harvested energy, even in a wireless power transfer scenario, due to the dynamics of the environment in which the signal propagates.

The TCTM problem seeks to determine an optimal packet scheduling scheme that minimizes the average delay experienced by all packets, thereby improving the quality of service of supported applications. Assuming random arrival of energy and data and the initial conditions of $E_{0}$ and $B_{0}$ of available energy and data, respectively. Let r and p represent data and power transmission, respectively, such that r=g(p) where g is the Shannon capacity function defined by $g\left(p\right)=\frac{1}{2}\log\left(1+p\right)$, the problem is formulated to find the optimum transmission power or rate that reduces the waiting time for a pre-known number of arriving packets.

Assuming that the transmitting WS adjusts its transmission power N times before the end of the transmission with a sequence $p_{1},\;p_{2},\;\ldots ,p_{N}$, and that the corresponding transmission times are, respectively $t_{1},\;t_{2},\ldots ,\;t_{N}$, the total energy consumed at a given moment t is defined in [106] as follows:

(23) $$E\left(t\right)=\overset{\bar{i}}{\underset{i=1}{\sum }}p_{i}t_{i}+p_{\bar{i}+1}\left(t-\overset{\bar{i}}{\underset{i=1}{\sum }}t_{i}\right)$$

where $\bar{i}=\max\left(i:\sum_{j=1}^{i}t_{j}\leq t\right)$. The total number of bits B(t) transmitted at time t is also defined by:

(24) $$B\left(t\right)=\overset{\bar{i}}{\underset{i=1}{\sum }}g\left(p_{i}\right)t_{i}+g\left(p_{\bar{i}+1}\right)\left(t-\overset{\bar{i}}{\underset{i=1}{\sum }}t_{i}\right)$$

where g is the Shannon capacity function. From Equations (23) and (24), the TCTM problem is then formulated as the following constrained optimization problem:

(25) $$\underset{p,t}{\min}T\;\;\;\;\;\;\;\;0\leq t\leq T$$

Subject to:

(26) $$\{\begin{array}{c} E\left(t\right)\leq \underset{i:s_{i}<t}{\sum }E_{i}\\ B\left(T\right)=B_{0} \end{array}$$

where $E_{i}$ is the amount of harvested energy at a time $s_{i}$, a theorem was formulated for this optimization problem to determine the conditions for an optimum transmission policy.

Although the design proposed in [106] is a good basis for designing RF-EH-WSs by minimizing the transmission time of all packets, it appears that two different packets experience different delays, and the average transmission time of the system is not minimized. This constraint is addressed in [107], where the second constraint defined in Equation (26) is transformed into two conditions, thus integrating the transmission delay of each packet. A Lagrangian function is then defined, and the Karush-Kuhn- Tucker (KKT) conditions are formulated to find the optimal transmission power that minimizes all packets’ total transmission time. The transmission duration is minimized of each packet, thus optimizing the average transmission time.

The first drawback to the two previous studies [106,107] is that they consider an unlimited battery size and data buffer. Another drawback is the assumption of stability of the optimum transmission power between energy and data arrival events. This has the effect of increasing the delay accumulation for some data packets. An improved algorithm proposed in [108] deals with this issue. In that study, the optimum transmission power starts high, decreases linearly, and potentially reaches zero between energy and data arrivals. For this purpose, the model of Figure 20 is adapted and considered for an additive white gaussian noise (AWGN) channel. The harvested energy $E_{m}$ is different at a different moment $t_{m}$, where m=0,1,…, M−1. The data to be transmitted also arrives with an amount of different sizes $B_{m}$. The maximum energy that can be stored in a battery is $E_{\max}$, and the data buffer size is also limited to a maximum capacity of $B_{\max}$. By defining the delay experienced by each bit as being the time interval between the moment of its arrival and the instant of its transmission, the average total delay of the system is defined as follows:

(27) $$\bar{D}=\overset{\infty }{\underset{0}{\int }}tdB\left(t\right)-\overset{\infty }{\underset{0}{\int }}tdB_{a}\left(t\right)$$

where B (t) is the total departed data to the receiver, and $B_{a}\left(t\right)$ is the total amount of received data at time t. The objective is to define the optimum power policy, which minimizes the average time. For a given data arrival scenario, the second term of Equation (27) is constant, which results in reducing only the raw delay defined as:

(28) $$D=\overset{\infty }{\underset{0}{\int }}tdB\left(t\right)=\overset{\infty }{\underset{0}{\int }}\frac{t}{2}\log\left(1+p\left(t\right)\right)dt$$

The optimization problem defined in [108] is then formulated as in Equations (29) and (30) where $E_{a}\left(t\right)$ is the cumulative harvested energy at time $t_{m}$, and m=1,…,M. To resolve this problem, a recursive solution is used to find the optimal transmission power over time by determining the right Lagrange multipliers.

(29) $$\underset{p}{\min}\overset{\infty }{\underset{0}{\int }}t\log\left(1+p\left(t\right)\right)dt$$

Subject to:

(30) $$\{\begin{array}{c} E_{a}\left(t_{m}\right)-E_{max}\leq \overset{t_{m}}{\underset{0}{\int }}p\left(t\right)dt\leq E_{a}\left(t_{m}\right)\\ B_{a}\left(t_{m}\right)-B_{max}\leq \overset{t_{m}}{\underset{0}{\int }}\log\left(1+p\left(t\right)\right)dt\leq B_{a}\left(t_{m}\right)\\ \overset{\infty }{\underset{0}{\int }}\log\left(1+p\left(t\right)\right)dt=B_{a}\left(t_{M}\right)\\ p\left(t\right)\geq 0,\;\;\;\;\forall t \end{array}$$

### 6.2. Short-Term Throughput Maximization (STTM) Problem

**Figure 21:**
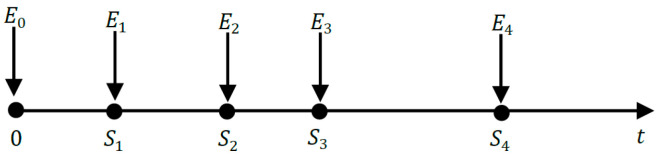
Energy harvesting communication model for solving STTM problem.

This main issue deals with maximizing the number of bits sent before the end of the transmission. The fundamental principle is the same as in the TCTM problem. It defines an allocation/transmission policy in which a maximum number of bits is transmitted for a given duration. This problem was first addressed in [109,110]. The most used model is shown in Figure 21. It is assumed that the WS can transmit with a finite level of power p(t) corresponding to an instantaneous rate r=g(p(t)) where g (.) is the Shannon Capacity function. The STTM focuses solely on energy harvesting and how to use this energy. The energy spent is strongly related to the energy used to transmit the data. The battery is supposed to be able to accumulate energy up to the maximum capacity $E_{\max}$. The overflowing energy is subsequently lost. Thus, the defined model takes care of two constraints resulting from the fact that at a given instant, the available energy is not enough. By contrast, extra energy is lost when the battery is fully charged. From these two constraints, a set of feasible power allocations has been defined in [110] as:

(31) $$ℬ=\left\{p\left(t\right)/0\leq \overset{n-1}{\underset{k=0}{\sum }}E_{k}-\overset{t^{\prime }}{\underset{0}{\int }}p\left(t\right)dt\leq E_{\max}\right\}\forall \;n>0,s_{n-1}\leq t^{\prime }<s_{n}$$

where $E_{k}$ is the energy harvested at the moment $S_{k}$ as illustrated in Figure 21. The optimization problem is then formulated as below; r(p(t)) is the power-rate function.

(32) $$\underset{p\left(t\right)}{\max}\overset{T}{\underset{0}{\int }}r\left(p\left(t\right)\right)dt\;,\;s.t.\;p\left(t\right)\in ℬ$$

The main objective of this section was to present techniques for maximizing energy efficiency in RF-EH-WSs. The leading solutions considered are the minimization of delays and maximization of throughput. In the following section, we present some avenues for future research for the problem under review.

## 7. Summary of the Main Results of This Review, Challenges, and Suggestions for Future Research

This review discussed four major axes to support the development foundations of RF energy harvesting for WS applications, namely the rectenna feeding techniques (RFT), design issues of rectennas, general solutions for minimizing the spent energy of the WS (MEB-WS), and efficient management of the harvested energy (PMM). At first glance, the challenge that emerges from this review is the possibility of *designing a miniaturized self-sufficient sensor node for WS and IoT applications*. In keeping with the previous sections, future perspectives for study are discussed in the following subsections. Optimization problems in the design will also be formulated to minimize the circuits’ volume.

### 7.1. In the Rectenna Feeding Techniques Field

**Figure 22:**
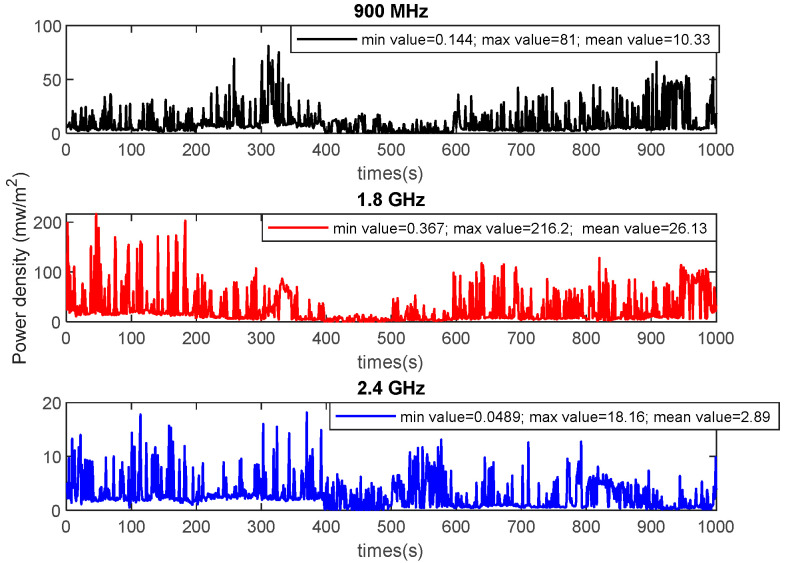
Variation in ambient power density in the 900 MHz, 1.8 GHz and 2.4 GHz bands.

Despite its ubiquity, the amount of harvestable RF power is very random, even in the case of wireless power transfer. For illustration, the power density measurements in three different frequency bands, 900 MHz, 1.8 GHz, and 2.4 GHz, are shown in Figure 22. The data is collected every second for about 16.5 min; these data are recorded with an Extech electric field measurer (the 8GHZ RF/EMF STRENGTH METER). This RF/EMF meter records electric field data for frequencies between 10 MHz and 8 GHz. The data were recorded between 10 a.m., and 10 past 17 min on 4 September at the department of industrial Electronics of the Cegep in Abitibi Temiscamingue.

These measurements show that deviations of 10.2, 216, and $18\;\mathrm{mW}/m^{2}$ are observable in the 900 MHz, 1.8 GHz, and 2.4 GHz frequency bands, respectively. The figures also show periods of low power densities. However, in most IoT applications, events can occur at any time (IoT application). These fluctuations will make it difficult to predict the performance of RF-EH-WSs in a real environment.

Therefore, we suggest analyzing the characteristics of the RF signals in greater depth to better exploit them. Suggested leads could be deep learning of signal propagation before WSs deployment or even using chaotic signal analysis techniques (for more complex and dynamic environments) to analyze RF signals.

### 7.2. In the Rectenna Design Field

- Considering the design equations of a patch antenna or DRA antenna, nonlinear optimization techniques could be used to size the antenna for miniature rectenna applications. The objective function could be the antenna size, and constraints would be set on a minimum gain to be achieved.
- For multi sources-harvesting or multi-band harvesting, smart antennas for the design of rectennas, such as switched beam antennas, can be considered [111]. In this regard, it would be helpful to propose an analytical study to justify the need for these antennas by quantifying the power consumed to make the antenna wise.
- Regarding the RF signal rectification, with the voltage doubler (the most efficient rectifier), it would be necessary to derive the equations that make it possible to analyze the different circuit losses (as a function of the electrical elements of the used diode) in this configuration. This would make it possible to propose new rectifying diodes that would be more efficient for the RF-EH process.

### 7.3. In the MEB-WS Field

Considering the current topology of IoT networks (a star network), the information routing techniques are outdated because each node communicates directly with the sink. It would be essential to intensify research on data compression techniques and channel access techniques for IoT or star networks. In this research, one could consider other network topologies, such as the cluster topology for remote communications in IoT networks. In this topology, the lower energy adaptive clustering hierarchy (LEACH) protocol makes it possible to envisage an equitable distribution of the network loads between the different sensors. It should be reconsidered, taking features related to the randomness of the RF source. More likely, the choice of cluster heads should be led by the rate of the energy harvesting process.

### 7.4. In the RF-EH-WS Field (PMM)

Future research could propose a global design strategy integrating rectenna and WS. Modeling the behavior of an RF-EH-WS would also be a significant advance for IoT networks. The study of the impact of the environment on the reliability of WS communication would also be interesting to evaluate the minimum quality of service that can be offered. Another research avenue would be to analyze the autonomy or profitability obtained with an RF-EH-WS system depending on the considered IoT application. The performance criteria can be the data rate and the transmission range of the autonomous WS.

## 8. Conclusions

Due to the operation of telecommunications equipment, RF energy has become ubiquitous, and these waves can travel in any environment. These two characteristics make the RF source the most popular primary source to ensure the energy autonomy of WSs in environments that are difficult to access. An RF-EH-WS will thus support the objectives of IoT, for which real-time communication is necessary everywhere. However, the operability of an RF-EH-WS results from efforts in several research fields. In this review, we proposed an overview of the design considerations of RF-EH-WSs. For this, this study breaks down an RF-EH-WS system into four subsystems that must be characterized to precisely define the specifications of the autonomous WS. Considered subsystems include the feeding technique (RFT), RF-EH system (Rectenna), WS energy budget minimization (MEB-WS), and finally, efficient management of the harvested energy. For each subsystem, the main characteristics are defined, and the constraints in terms of the sizes of the used components are described.

Regarding RFTs, ambient RF energy harvesting (A-RF-EH) is distinguished from wireless power transfer (WPT). Regarding A-RF-EH, the power density levels measured in recent years are reported. For WPT, the propagation models proposed to evaluate the signal attenuation are reviewed for each technique mentioned. The different blocks of a rectenna (antenna, RF/DC conversion, matching network, and DC/DC converter) are examined in terms of conversion efficiency. In addition to theoretical knowledge, the characteristics of the main commonly used components are highlighted for each of these blocks. Following this, we provided the factors that influence the energy consumption of the WS. While defining each of these aspects, it is seen that the energy budget of a WS will depend on its hardware architecture, the topology of the network that uses it, and finally, its communication protocol. Ultimately, we defined the techniques used to optimize the energy efficiency of an RF-EH-WS. It follows from the state of the art that the proposed solutions either maximize the throughput or minimize the transmission delays. This study also identifies imminent research to design miniaturized and energy-self-sufficient RF-EH-WSs to support IoT applications. Considering all the aspects mentioned above, this paper lays the foundation for new RF-EH-WS designs optimized to achieve the performance predefined by the amount of harvestable RF energy.

## Figures and Tables

**Table 1:** A summary of the state-of-the-art surveys on RF-EH-WS.

Papers	RFT	RF-EH	MEB-WS	RF-EH-WS	Key Points
[14] (2007)	×	×	×	√	Harvesting theory Design implications and examples Power management algorithms
[15] (2011)	×	×	×	√	Energy harvesting architectures Main primary energy sources Storage technologies
[21] (2014)	√	√	×	×	Energy-harvesting circuit characterization State-of-the-art RF energy harvesters
[18] (2014)	√	√	×	×	WPT techniques The architecture of RF-EH networks SWIPT beamforming
[22] (2015)	√	√	×	√	Classification of RF energy harvesting Overview of RF-powered cognitive radio network Technical challenges of RF-powered cognitive radio network
[19] (2016)	×	×	×	√	Energy sources models Energy harvesting models
[17] (2016)	√	√	×	√	Rectenna design and optimization Communications analysis RFID transponder Modulation and demodulation
[20] (2018)	√	√	×	×	WPT SWIPT emerging technologies for fifth-generation communications
[27] (2019)	√	×	×	×	Investigation on different RF energy sources for RF energy harvesting
[26] (2021)	×	√	×	×	Rectenna applications Single band and dual band rectennas for wireless applications Various techniques adopted for circular polarization
[9] (2022)	√	√	×	×	RF-EH applications RF concept and principles Power -harvesting evaluation metrics
[25] (2022)	√	√	×	×	Harvesting methods Application of RF energy harvesting RF energy harvesting evaluation metrics
This review (2022)	√	√	√	√	Rectenna feeding techniques MEB-WS by network topology Rectenna optimization solution Optimization of energy efficiency by maximizing throughput and minimizing delays

**Table 2:** Various ambient RF power levels.

Ref	Bands	Frequency (MHz)	Average Power Densities (nW/cm^2^)	Measured Power (μW)	City/Country
[31]	Professional mobile radio	415–425	$2.3\times 10^{5}$	-	Zagreb/Croatia
	DTV	470–790	$3.78\times 10^{6}$	-	Croatia
[28]	DTV (during switch over)	470–610	8.9	-	London/UK
	GSM 900 (MTX)	880–915	$4.5\times 10^{-7}$		
	GSM 900 (BTX)	925–960	36		
	GSM 1800 (MTX)	1710–1785	0.5		
	GSM 1800 (BTX)	1805–1880	84		
	3G (MTX)	1920–1980	0.46		
	3G (BTX)	2110–2500	0.18		
	Wi-Fi	2400–2500	12		
[32]	Wi-Fi	2400		630	Val d’Or/Canada
[33]	GSM 900/LTE Band 8, GSM 1800/ LTE Band 3, UMTS Band 1, ISM Wi-Fi 2.4 GHz, LTE Band 7	900–3000	-	63.1	Paris/France
[34]	LTE 700 MHz, GSM 850 MHz, ISM 900 MHz	700/850/900	-	3.2	Boston/USA
[35]	CDMA downlink	870–880	-	0.126	Shunde/China
	GSM 900	935–960	-	0.01	

**Table 3:** Commonly used Schottky diodes in RF-EH system designs.

Diodes	SMS 7630 (Skyworks)	SMS 7621 (Skyworks)	SMS 1546 (Skyworks)	HSMS 2820 (Avago)	HSMS 2850 (Avago)	HSMS 2860 (Avago)	MA4E 1317 (Macon)	MA4E 2054 (Macon)
$R_{S}\left(\Omega \right)$	20	12	4	6	25	5	4	11
$V_{j}\;\left(V\right)$	0.34	0.51	0.51	0.65	0.35	0.65	0.7	0.4
$C_{j_{0}}\left(\mathrm{pF}\right)$	0.14	0.1	0.38	0.7	0.18	0.18	0.2	0.13
$V_{B}\left(V\right)$	1	2	2	15	2	7	7	3

**Table 4:** Features of some commercialized MEMS sensors.

Component	Manufacturer	Sensor Type	Supply (V)	Consumption (mA)
CXL04GP3	Aceinna	Accelerometer	4.9–5.5	3
ADXL278	Analog Devices	Accelerometer	4.75–5.25	2.2
ADXL325	Analog Devices	Accelerometer	1.8–3.6	0.35
MPL115A	Freescale	Pressure	3.3–5.5	0.005
DTH22	Adafruit	Temperature and humidity	3.3–6	1.5
STLM20	ST	Temperature	2.4–5.5	0.008

**Table 5:** Features of some low power marketed microprocessors.

Component	Manufacturer	Supply (V)	Sleep (μA)	Processing (mA)	Receive (mA)	Transmit (mA)
ATMega128	Atmel	2.7	15	8	19.7	17.4
MSP430F5437	Texas Instrument	2.2–3.6	12	2.2	18.5	18.5
MSP430L092	Texas Instrument	0.9–1.65	6	0.18	-	-
MSP430G2553	Texas Instrument	1.8–3.6	0.5	0.23	-	-
ARM920T	ARM	4.5–5.5	33	104	40	40
ATmega1281	Atmel	3.3–4.2	55	15	30	30
Marvell PXA271	Marvell	3.2	390	31–53	44	44

**Table 6:** Features of some commercial radio chip for WS.

Component	Manufacturer	Supply (V)	Sleep (μA)	Receive (mA)	Transmit (mA)	Maximum Transmission Power (dBm)
CC2430	Texas Instrument	2–3.6	0.5	27	27	0
CC2590	Texas Instrument	2.2–3.6	0.1	34	22.1	12.2
CC2520	Texas Instrument	1.8–3.8	1	18.5	33.6	5
TCM 300	EnOcean	2.6–4.5	-	33	24	5
EM250	Ember	2.1–3.6	1	29	33	5
nRF24AP2	Nordic	1.9–3.6	0.5	17	15	0
JN5139	Jennic	2.2–3.6	0.2	34	35	3
SX1211	Semtech	2.1–3.6	2	3	25	10
MC1321	Freescale	2–3.4	1	37	30	0

## Data Availability

Not applicable.
